# Combinations of Host- and Virus-Targeting Antiviral Drugs Confer Synergistic Suppression of SARS-CoV-2

**DOI:** 10.1128/spectrum.03331-22

**Published:** 2022-10-03

**Authors:** Jessica Wagoner, Shawn Herring, Tien-Ying Hsiang, Aleksandr Ianevski, Scott B. Biering, Shuang Xu, Markus Hoffmann, Stefan Pöhlmann, Michael Gale, Tero Aittokallio, Joshua T. Schiffer, Judith M. White, Stephen J. Polyak

**Affiliations:** a Virology Division, Department of Laboratory Medicine and Pathology, University of Washington, Seattle, Washington, USA; b Department of Immunology, University of Washington, Seattle, Washington, USA; c Institute for Molecular Medicine Finland (FIMM), HiLIFE, University of Helsinki, Helsinki, Finland; d Division of Infectious Diseases and Vaccinology, School of Public Health, University of California—Berkeley, Berkeley, California, USA; e Vaccine and Infectious Diseases Division, Fred Hutchinson Cancer Research Center, Seattle, Washington, USA; f Infection Biology Unit, German Primate Center, Leibniz Institute for Primate Research, Göttingen, Germany; g Faculty of Biology and Psychology, University of Göttingen, Göttingen, Germany; h Oslo Centre for Biostatistics and Epidemiology, University of Oslo and Oslo University Hospital, Oslo, Norway; i Division of Allergy and Infectious Disease, University of Washington, Seattle, Washington, USA; j Clinical Research Division, Fred Hutchinson Cancer Research Center, Seattle, Washington, USA; k Department of Cell Biology, University of Virginia, Charlottesville, Virginia, USA; l Department of Microbiology, University of Virginia, Charlottesville, Virginia, USA; m Department of Global Health, University of Washington, Seattle, Washington, USA; n Department of Microbiology, University of Washington, Seattle, Washington, USA; Fundacio irsiCaixa

**Keywords:** SARS-CoV-2, coronavirus, host-targeting antiviral, HTA, directly acting antiviral, DAA, combination, synergy, molnupiravir, camostat, paxlovid, Calu-3, antiviral, drug combinations, variant(s) of concern, Ebola

## Abstract

Three directly acting antivirals (DAAs) demonstrated substantial reduction in COVID-19 hospitalizations and deaths in clinical trials. However, these agents did not completely prevent severe illness and are associated with cases of rebound illness and viral shedding. Combination regimens can enhance antiviral potency, reduce the emergence of drug-resistant variants, and lower the dose of each component in the combination. Concurrently targeting virus entry and virus replication offers opportunities to discover synergistic drug combinations. While combination antiviral drug treatments are standard for chronic RNA virus infections, no antiviral combination therapy has been approved for SARS-CoV-2. Here, we demonstrate that combining host-targeting antivirals (HTAs) that target TMPRSS2 and hence SARS-CoV-2 entry, with the DAA molnupiravir, which targets SARS-CoV-2 replication, synergistically suppresses SARS-CoV-2 infection in Calu-3 lung epithelial cells. Strong synergy was observed when molnupiravir, an oral drug, was combined with three TMPRSS2 (HTA) oral or inhaled inhibitors: camostat, avoralstat, or nafamostat. The combination of camostat plus molnupiravir was also effective against the beta and delta variants of concern. The pyrimidine biosynthesis inhibitor brequinar combined with molnupiravir also conferred robust synergistic inhibition. These HTA+DAA combinations had similar potency to the synergistic all-DAA combination of molnupiravir plus nirmatrelvir, the protease inhibitor found in paxlovid. Pharmacodynamic modeling allowed estimates of antiviral potency at all possible concentrations of each agent within plausible therapeutic ranges, suggesting possible *in vivo* efficacy. The triple combination of camostat, brequinar, and molnupiravir further increased antiviral potency. These findings support the development of HTA+DAA combinations for pandemic response and preparedness.

**IMPORTANCE** Imagine a future viral pandemic where if you test positive for the new virus, you can quickly take some medicines at home for a few days so that you do not get too sick. To date, only single drugs have been approved for outpatient use against SARS-CoV-2, and we are learning that these have some limitations and may succumb to drug resistance. Here, we show that combinations of two oral drugs are better than the single ones in blocking SARS-CoV-2, and we use mathematical modeling to show that these drug combinations are likely to work in people. We also show that a combination of three oral drugs works even better at eradicating the virus. Our findings therefore bode well for the development of oral drug cocktails for at home use at the first sign of an infection by a coronavirus or other emerging viral pathogens.

## INTRODUCTION

Despite the approval of one intravenous (remdesivir) and two oral (molnupiravir and paxlovid) anti-SARS-CoV-2 drugs, the armamentarium against SARS-CoV-2 and related coronaviruses remains thin. Monoclonal antibodies have proven effective as prophylaxis and therapy (1, 2) but must be given intravenously or subcutaneously in a clinical setting. Initially effective products have rapidly become irrelevant in the face of newly emerging viral variants (3). While highly effective at preventing severe disease if given early during infection in outpatient settings (4), remdesivir has been mostly given during hospitalization (5, 6). Its use during early disease is limited due to its intravenous formulation. Molnupiravir, an oral drug, is 30% effective at preventing hospitalization when given early during infection (7). Paxlovid, another oral drug, is highly efficacious (8), but side effects, drug interactions (9), and rebound of symptoms and viral shedding upon treatment cessation (10, 11) can limit therapy success. The recent descriptions of paxlovid resistance *in vitro* (12–16) suggests there may be at some point no highly effective oral agent to prevent hospitalization in high-risk infected people. Thus, there remains a need to develop SARS-CoV-2 prophylactic and therapeutic regimens that are suitable for self/home administration, via inhaled or oral routes, particularly for infected people who are newly diagnosed and early in their COVID-19 course (17). Ideally, these treatments should be relatively inexpensive so they can be widely used by infected people and their contacts around the globe.

It is well established that the most effective drug-based therapies for chronic, persistent RNA viruses include a combination of two or three drugs (18, 19). Drug combinations comprise the standard of care for chronic RNA virus infections such as HIV and HCV infections and demonstrate promise for treating acute RNA virus infections, including infections with filoviruses (20–22), arenaviruses (23), influenza viruses (24–27), and, most recently, SARS-CoV-2 (28–31; for a review, see reference 32). A key feature of drug combinations is that they may confer bioactivities beyond additivity, such as multiplicative or synergistic effects, by targeting different steps of the viral life cycle. (Paxlovid is a combination treatment consisting of nirmatrelvir [PF-07321332], which inhibits the SARS-CoV-2 3C-like protease, and ritonavir, which slows the metabolism of nirmatrelvir [33]. However, paxlovid does not leverage dual drug target sites.) Synergistic antiviral activity may permit dose reductions of each drug in the combination, thereby reducing the potential for clinical side effects while allowing increased clinical efficacy. Moreover, synergistic drug combinations can bring *in vitro* drug levels that inhibit virus by 50% (IC_50_) into *in vivo* pharmacokinetic (PK) ranges. For chronic RNA virus infections such as HIV and HCV infections, drug combinations critically limit emergence of drug resistant viral mutants (18, 19).

Combination therapies may be of particular use for immunocompromised patients with SARS-CoV-2 infection. More potent treatments are likely needed for patients who may not be able to exert adequate immune pressure on the virus (34–36). Moreover, SARS-CoV-2 evolution in immunosuppressed patients has become a major public health concern since variants of concern (VOCs) may arise during SARS-CoV-2 infections that persist at high viral load for months (37). Novel VOC continue to extend the pandemic by undermining vaccine efforts. There is also a greater window for the emergence of drug-resistant mutants in this setting.

Oral drug combination regimens are often preferred to allow outpatient treatment early during disease as prompt treatment after symptom onset is associated with better outcomes for SARS-CoV-2 virus (1, 2, 4, 7), influenza virus (38), Ebola virus (39), HIV (40), and zoster virus (41) infections. Here, we demonstrate the synergistic potential of combining host-targeting antivirals (HTAs) that either target SARS-CoV-2 entry (camostat, nafamostat, and avoralstat) or an HTA (brequinar) that targets SARS-CoV-2 replication with a DAA replication inhibitor (molnupiravir) to block SARS-CoV-2 infection in Calu-3 lung epithelial cells. All four agents (three oral and one inhaled) synergized with the oral agent molnupiravir, with the pairs camostat plus molnupiravir and brequinar plus molnupiravir demonstrating the strongest synergy, which was similar to the synergy observed with the all-DAA combination of molnupiravir plus nirmatrelvir. When the three component drugs, brequinar, camostat, and molnupiravir were combined, even greater antiviral efficacy and potency was observed. Pharmacodynamic (PD) modeling of the *in vitro* data suggest drug concentrations at which high potency can be achieved, which may fall within observed plasma values of these agents, hence suggesting possible *in vivo* effects.

## RESULTS

### Choice of drugs for combination testing versus SARS-CoV-2.

We aim to develop a combination of oral or inhaled drugs with potent activity against SARS-CoV-2 in lungs. Towards this end, we explored combined targeting of SARS-CoV-2 entry and replication, as blocking different phases of the SARS-CoV-2 life cycle offers the possibility of synergistic antiviral efficacy (32). Our initial focus drugs targeted TMPRSS2, a host cell serine protease critical for SARS-CoV-2 entry into lung cells (42), and molnupiravir, a potent oral inhibitor of the SARS-CoV-2 RNA-dependent RNA polymerase (43). The TMPRSS2 inhibitors included two approved drugs, camostat and nafamostat, as well as the preclinical drug avoralstat (42, 44, 45). Camostat and avoralstat are both oral drugs, while nafamostat is administered intravenously and is being tested via inhalation for COVID-19. All three drugs have shown efficacy in small animal models of SARS-CoV-2 infection via oral or inhaled routes (45, 46). Both oral camostat and intravenous nafamostat have been studied in small SARS-CoV-2 treatment trials in hospitalized patients, but they have shown no or only small benefits as solo agents (47–49). We considered three additional oral drugs proposed as entry inhibitors: apilimod, arbidol, and imatinib (50–53). However, based on literature values and our studies with VSV pseudovirions expressing the SARS-CoV-2 spike (S) protein, these oral drugs had lower bioavailability (*C*_max_) compared to their concentration for 50% inhibition (IC_50_) in lung cells than either camostat or avoralstat (see Table S2) and so were not pursued here. In addition to molnupiravir, we added brequinar to our set of drugs for combination testing. Brequinar is a pyrimidine biosynthesis inhibitor that one of us previously showed to synergize with the HCV polymerase inhibitor sofosbuvir to thwart HCV infection (29, 54), and it was also recently shown to synergize with molnupiravir against SARS-CoV-2 (31). As for other cell-based tests of molnupiravir, we employed its active form, EIDD-1931, for our studies.

**FIG 1:**
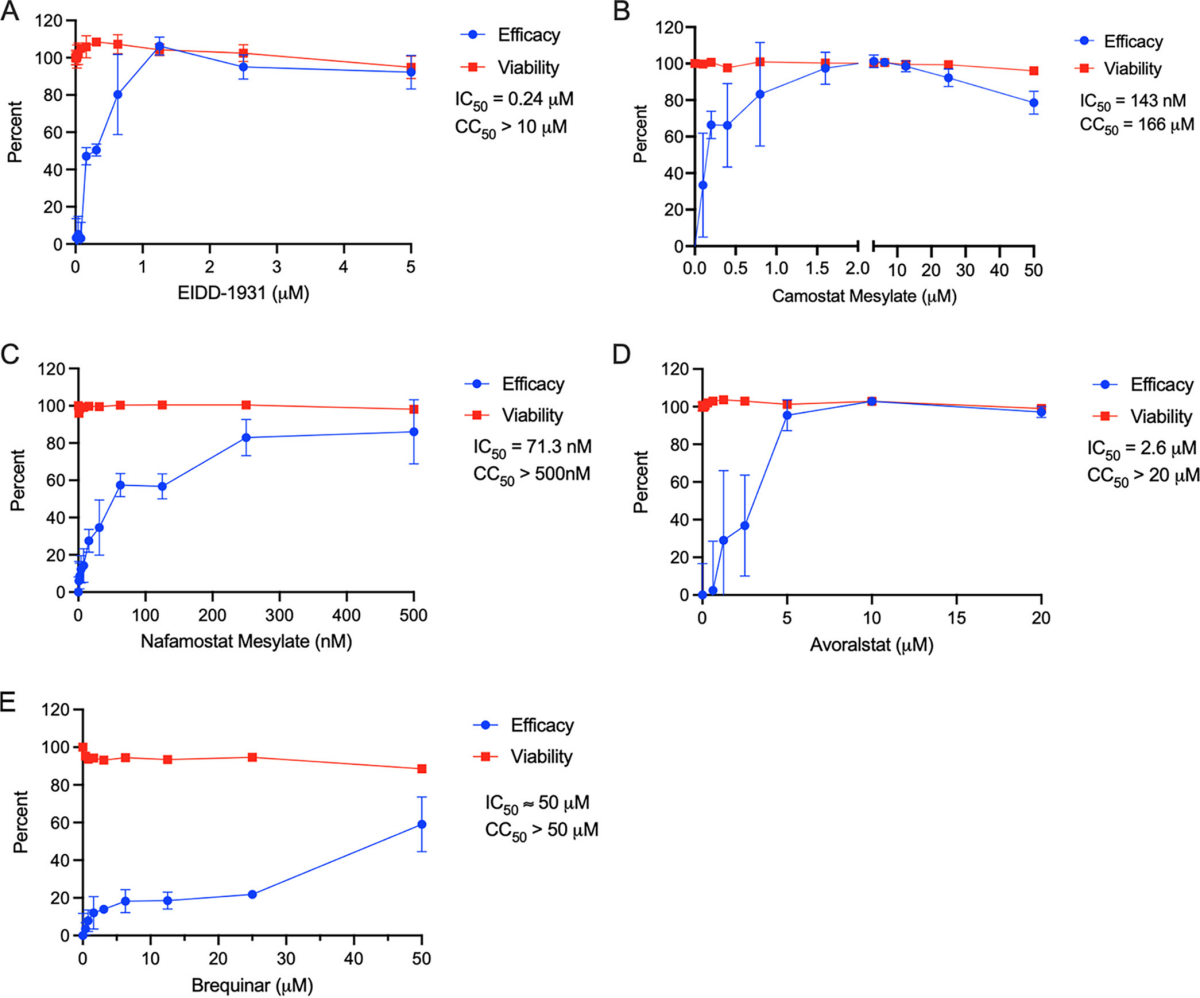
Inhibition of SARS-CoV-2 infection by DAA and HTA agents. Calu-3 cells were treated with the indicated concentrations of drugs for 2 h prior to infection with SARS-CoV-2 WA1 at an MOI of 0.1. Parallel plates contained cells treated only with drugs to monitor the toxic effects in noninfected cells (viability trace). At 96 h postinfection (72 h for camostat), the cell viability was assessed using a CellTiter-Glo assay, and the antiviral efficacy and viability, expressed as percentages relative to the DMSO solvent control, were calculated as described in Materials and Methods. Data points reflect averages and standard deviations of triplicate samples per condition, and IC_50_ and CC_50_ values were generated by nonlinear regression using Prism. The data represent an independent experiment for each drug. Note that for brequinar, we observed variable IC_50_s (22.4 μM ± 24, *n* = 3).

We first established antiviral dose responses for the compounds as single agents. EIDD-1931, camostat, nafamostat, avoralstat, and brequinar yielded IC_50_s against SARS-CoV-2 of 240 nM, 143 nM, 71.3 nM, 2.6 μM, and 50 μM, respectively, in Calu-3 cells (Fig. 1) with minimal toxicity on noninfected cells over the dose ranges studied. To study the cell line specificity of the activities, we also evaluated molnupiravir, camostat, and avoralstat against SARS-CoV-2 infection of a 293T cell line that overexpresses ACE2 and TMPRSS2 (55). In these 293TAT cells, the IC_50_s against SARS-CoV-2 were 150 nM, 1.2 μM, and 1.4 μM for EIDD-1931, camostat, and avoralstat, respectively, similar to their potencies in Calu-3 cells with the exception of camostat, which was more active in Calu-3 compared to 293TAT cells (see Fig. S1 in the supplemental material).

### Combination testing identifies drug pairs with synergistic activity against SARS-CoV-2 in Calu cells.

**FIG 2:**
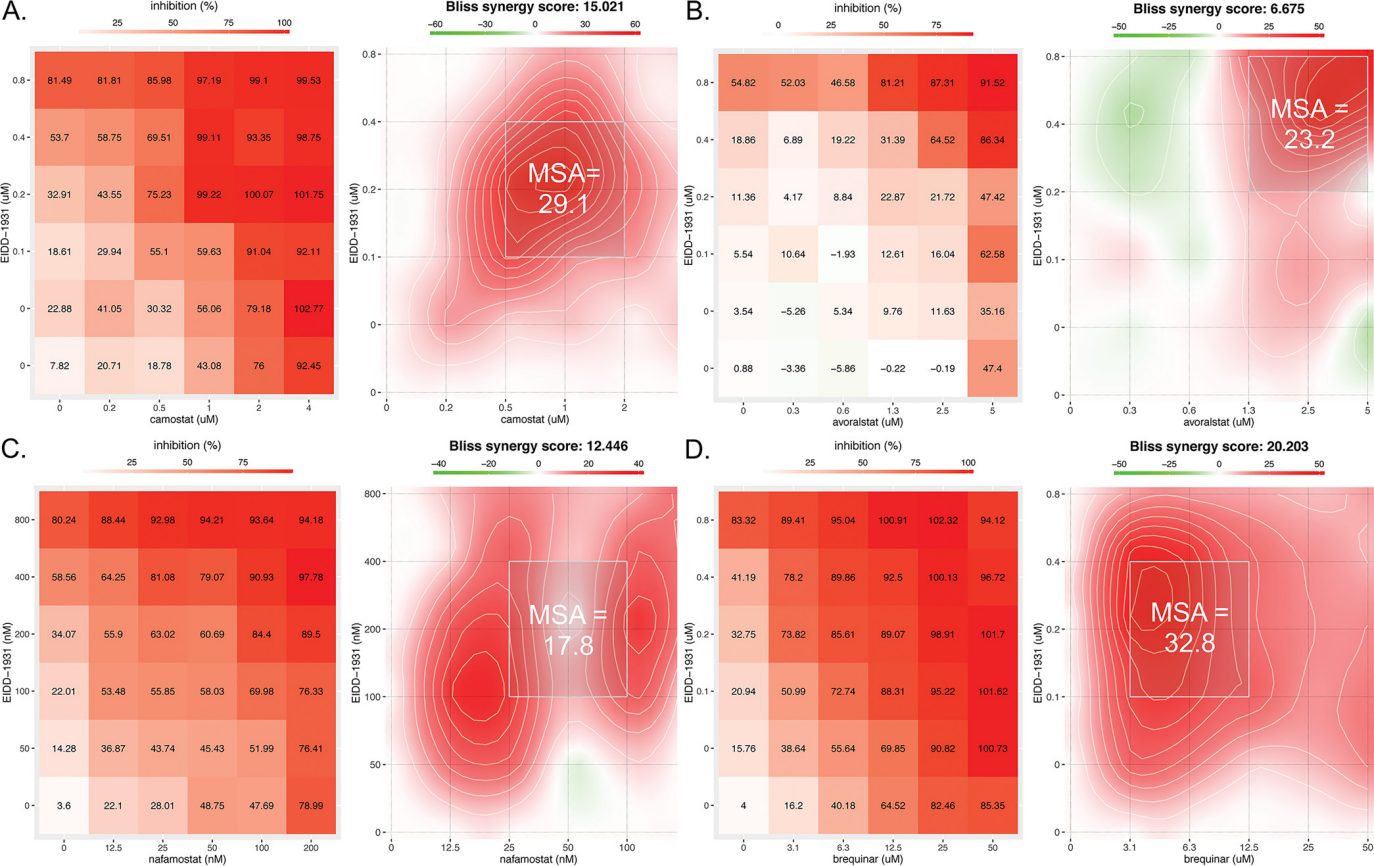
TMPRSS2 inhibitors and brequinar synergize with molnupiravir to suppress SARS-CoV-2 infection in Calu-3 cells. Calu-3 lung cells were treated with the indicated concentrations of camostat, avoralstat, nafamostat, brequinar, and molnupiravir (EIDD-1931) 2 h prior to infection with SARS-CoV-2 WA1 (MOI of 0.1). After 96 h, the cell viability was measured by a CellTiter-Glo assay (Promega), and the antiviral efficacy was calculated as described in Materials and Methods. For each panel (A through D), the left plot shows the percent inhibition of infection, while the right plot depicts a two-dimensional topograph that highlights the areas of synergy across the full dose response matrix, including the MSA, which is designated by a light gray box. Avoralstat induces maximal synergy at high concentrations of both drugs, whereas the other three compounds induce synergy at lower concentrations of both drugs.

Checkerboard drug combination assays were then performed with the three TMPRSS2 inhibitors and brequinar, each in combination with molnupiravir, first in Calu-3 cells. Figure 2 presents representative results as analyzed by SynergyFinder 3.0 (56). All drug combinations conferred dose-dependent synergistic suppression of virus infection. For the combinations of camostat plus molnupiravir, nafamostat plus molnupiravir, and brequinar plus molnupiravir, synergistic suppression of virus infection occurred at 2- to 3-fold lower drug concentrations for the combination compared to single drugs. The observed synergies boost drug effectiveness beyond what would be projected by Bliss independence. For example, for the combination molnupiravir plus camostat, each drug alone maximally provided 81 and 92% inhibitions of infection, respectively. However, the combination provided 99 to 100% inhibition in multiple dose combinations (Fig. 2A). When combined with molnupiravir, camostat and avoralstat showed similar synergistic suppression of SARS-CoV-2 infection of 293TAT cells (Table 1; see also Fig. S3). Thus, the combination effects of the TMPRSS2 inhibitors with molnupiravir extend to multiple human cell lines where virus entry occurs by TMPRSS2-mediated fusion. Table 1 summarizes the data from all drug pair experiments. None of the drug combinations in the checkerboard were toxic to noninfected cells, as assessed using SynToxProfiler (57) (see Fig. S2).

**TABLE 1:** Summary of combination data against SARS-CoV-2^*a*^

Molnupiravir +	Cells	Overall Bliss synergy	MSA	*N*
Camostat	Calu-3	13.2	26.0	2
Avoralstat	Calu-3	5.1	16.8	2
Nafamostat	Calu-3	8.7	15.0	2
Brequinar	Calu-3	22.6	33.4	2
Camostat	293TAT	15.6	21.3	2
Avoralstat	293TAT	10.7	15.6	2
Nirmatrelvir	Calu-3	13.0	23.8	3

a The overall Bliss synergy represents the average score for the entire 6 × 6 drug combination matrix, while maximum synergistic area (MSA) represents the score for a 3 × 3 submatrix. “*N*” refers to the number of separate experiments (biological replicates). For Calu-3 cells, all conditions in the checkerboard in each experiment were performed in triplicate, while for HEK293T cells overexpressing TMRPSS2 and ACE2 (293TAT), all conditions in the checkerboard were performed in duplicate in each experiment. Synergy scores and MSAs represent the averages of the replicated experiments. For all drug combinations, WA1 was the challenge virus, except for nirmatrelvir plus molnupiravir, where the delta VOC was the challenge virus.

Consistent with a recent report (31), combining brequinar with molnupiravir conferred robust, synergistic suppression of SARS-CoV-2 infection in Calu-3 cells (Fig. 2D and Table 1), with average synergy and MSA scores of 22.6 and 33.4, respectively. Synergy occurred at low to moderate drug concentrations, leading to high potency in these regions of the matrix. In these experiments, molnupiravir and brequinar maximally provided ~80% inhibition of infection as solo agents, whereas when applied together they provided 95 to 100% inhibition at multiple combinations of the drugs (Fig. 2D).

**FIG 3:**
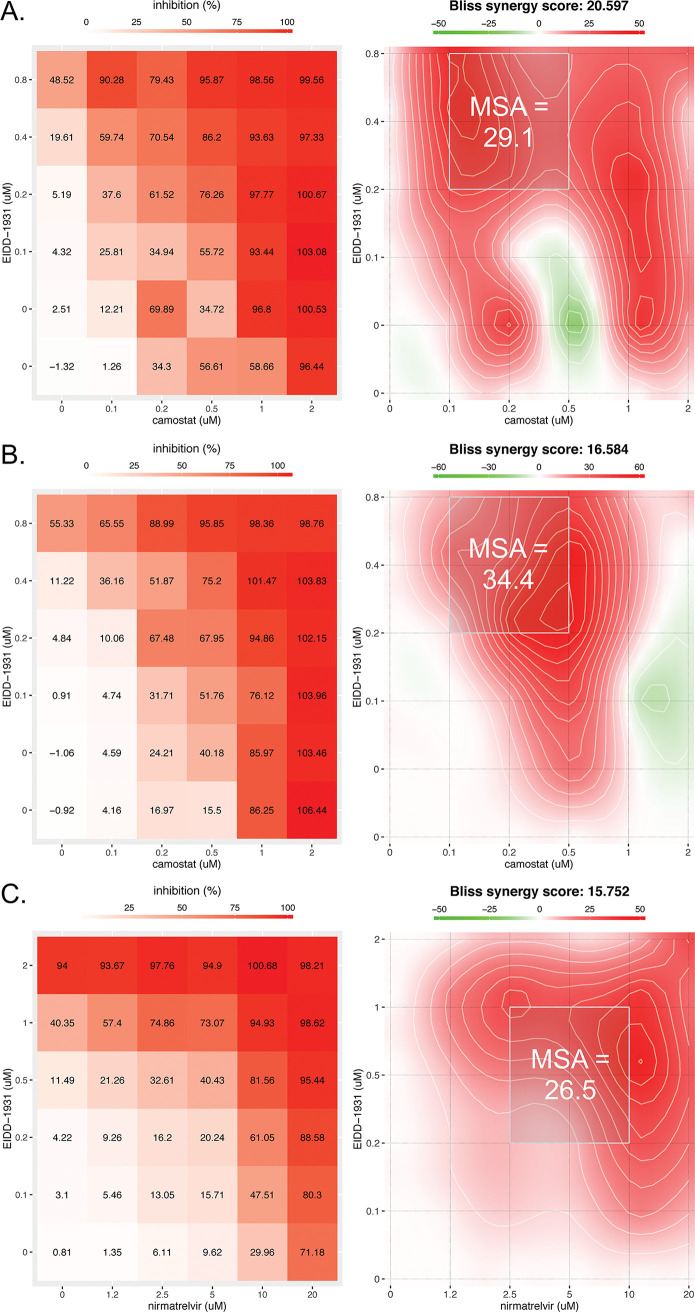
Camostat and nirmatrelvir synergize with molnupiravir to suppress infection of Calu-3 cells by SARS-CoV-2 VOCs. Calu-3 lung cells were treated with the indicated concentrations of camostat and molnupiravir (EIDD-1931) 2 h prior to infection with the SARS-CoV-2 beta (A) or delta (B) VOC at an MOI 0.01. (C) Nirmatrelvir and EIDD-1931 were added to Calu-3 cells 2 h before infection with the delta VOC at an MOI 0.01. After 96 h, the cell viability was measured by a CellTiter-Glo assay (Promega), and the antiviral efficacy was calculated as described in Materials and Methods. For each panel (A, B, and C), the left plot shows the percent inhibition of infection, while the right plot depicts a two-dimensional topograph that highlights the areas of synergy across the dose response matrix, including the MSA (light gray box).

To study whether the combinations show activity also against other variants, we next tested the combination of camostat plus molnupiravir against infection of Calu-3 cells by the beta and delta VOCs. This HTA+DAA combination conferred synergistic suppression of both VOCs (Fig. 3A and B). These combination effects were similar to the all-DAA combination of molnupiravir plus nirmatrelvir (Fig. 3C and Table 1).

### Predictive modeling of drug combination potency against SARS-CoV-2 in Calu-3 cells.

To recapitulate observed experimental data and then make projections about combinatorial drug potency at concentrations that were not measured experimentally, we applied an *in vitro* PD model previously validated against antiviral drug pairs (58). The model was tested against all four anti-SARS-CoV-2 DAA+HTA drug pairs in the present study: molnupiravir plus camostat, molnupiravir plus brequinar, molnupiravir plus avoralstat, and molnupiravir plus nafamostat. The model was assessed for fit to the empirically derived drug matrices in which percent inhibition of infection was assessed at various drug concentrations (left plots in Fig. 2A to D).

The mathematical model demonstrated high predictive power by closely projecting the *in vitro* efficacy of drug combinations across the dose response matrices, with an *R*^2^ value of ≥0.945 (Table 2). Exponent “*a*” is an approximation for overall synergy across dose response matrices. The drug pair molnupiravir plus brequinar has the highest exponent, indicating most synergistic interaction between the two drugs on average compared to other drug pairs, which is consistent with its highest MSA score (Fig. 2) among the DAA+HTA pairs tested. The model suggests that brequinar leads to synergy by lowering IC_50_ of molnupiravir more than camostat, avoralstat, or nafamostat (Table 2); e.g., the IC_50_ for molnupiravir in the brequinar combination is 0.09877 μM, whereas it is 0.1383, 0.1883, and 0.2866 μM, respectively, in the camostat, avoralstat, and nafamostat combinations.

**TABLE 2:** PD model parameters

Parameter^*a*^	Estimated value (95% CI)			
	Molnupiravir + camostat	Molnupiravir + brequinar	Molnupiravir + avoralstat	Molnupiravir + nafamostat
*a*	1.787 (0.7468–2.827)	3.85 (2.923–4.778)	3.314 (0.9883–5.639)	1.061 (0.6793–1.443)
*h*_EIDD_	1.139 (0.8407–1.437)	1.001 (0.8819–1.12)	1.181 (0.8145–1.548)	1.026 (0.8033–1.249)
*h*_2_	1.662 (1.257–2.066)	0.7289 (0.6431–0.8146)	1.748 (1.307–2.188)	0.9372 (0.7622–1.112)
IC_50_ (μM)				
IC_50,EIDD_	0.1383 (0.05129–0.2253)	0.09877 (0.07293–0.1246)	0.1883 (0.04864–0.328)	0.2866 (0.1662–0.4071)
IC_50,2_	0.533 (0.3205–0.7456)	2.858 (1.731–3.985)	1.265 (0.7121–1.819)	0.04567 (0.02515–0.0662)
*R* ^2^	0.9523	0.9851	0.945	0.959

a *h*_2_ and IC_50,2_ represent the Hill coefficient and the IC_50_ of camostat, brequinar, avoralstat, and nafamostat, respectively, for each drug combination.

**FIG 4:**
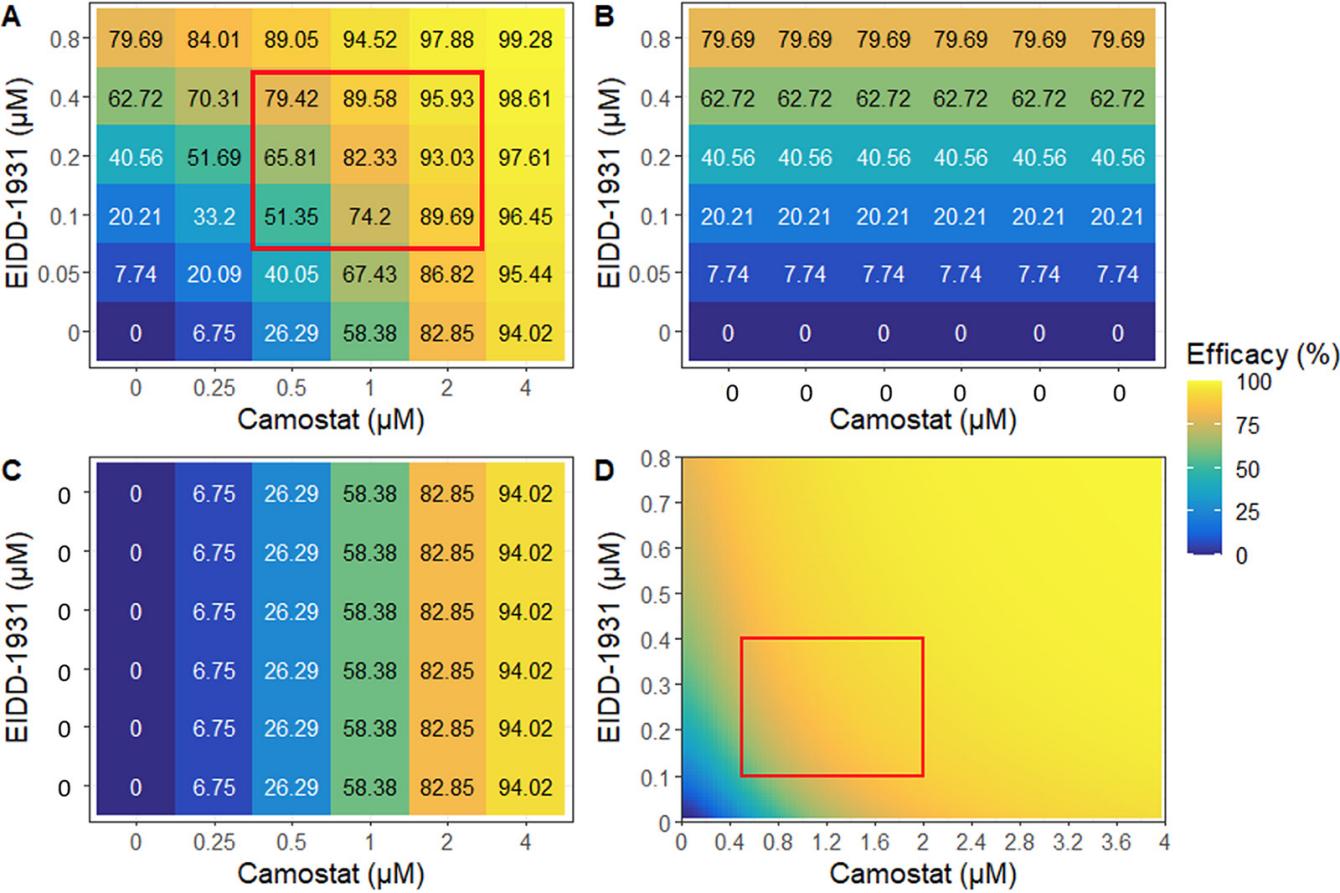
PD modeling of molnupiravir plus camostat. (A) Model-projected efficacy of molnupiravir (EIDD-1931) plus camostat at empirically tested concentrations. (B) Projected efficacy of EIDD-1931 alone. (C) Projected efficacy of camostat alone. (D) Heat map of model projected inhibition at all combinatorial concentrations of both agents. The red box denotes the MSA.

We next explored the mechanisms explaining the model fit to the data by focusing on individual drug combinations. For molnupiravir plus camostat, each drug lacked complete potency when modeled alone even at high concentrations (79.69% inhibition for molnupiravir [Fig. 4B] and 94.02% inhibition for camostat [Fig. 4C]). However, low concentrations of molnupiravir significantly boosted potency to >94% in the presence of high concentrations of camostat (4 μM, Fig. 4D). This enhanced potency is largely attributable to Bliss independence (i.e., multiplicative effects). Synergy boosts combinatorial effectiveness beyond predicted multiplicative effects at lower concentrations of camostat, particularly at 0.25 μM (Fig. 4D). Moreover, efficacies approaching 100% are observed across a large swath of the dose matrix (Fig. 4D, yellow).

**FIG 5:**
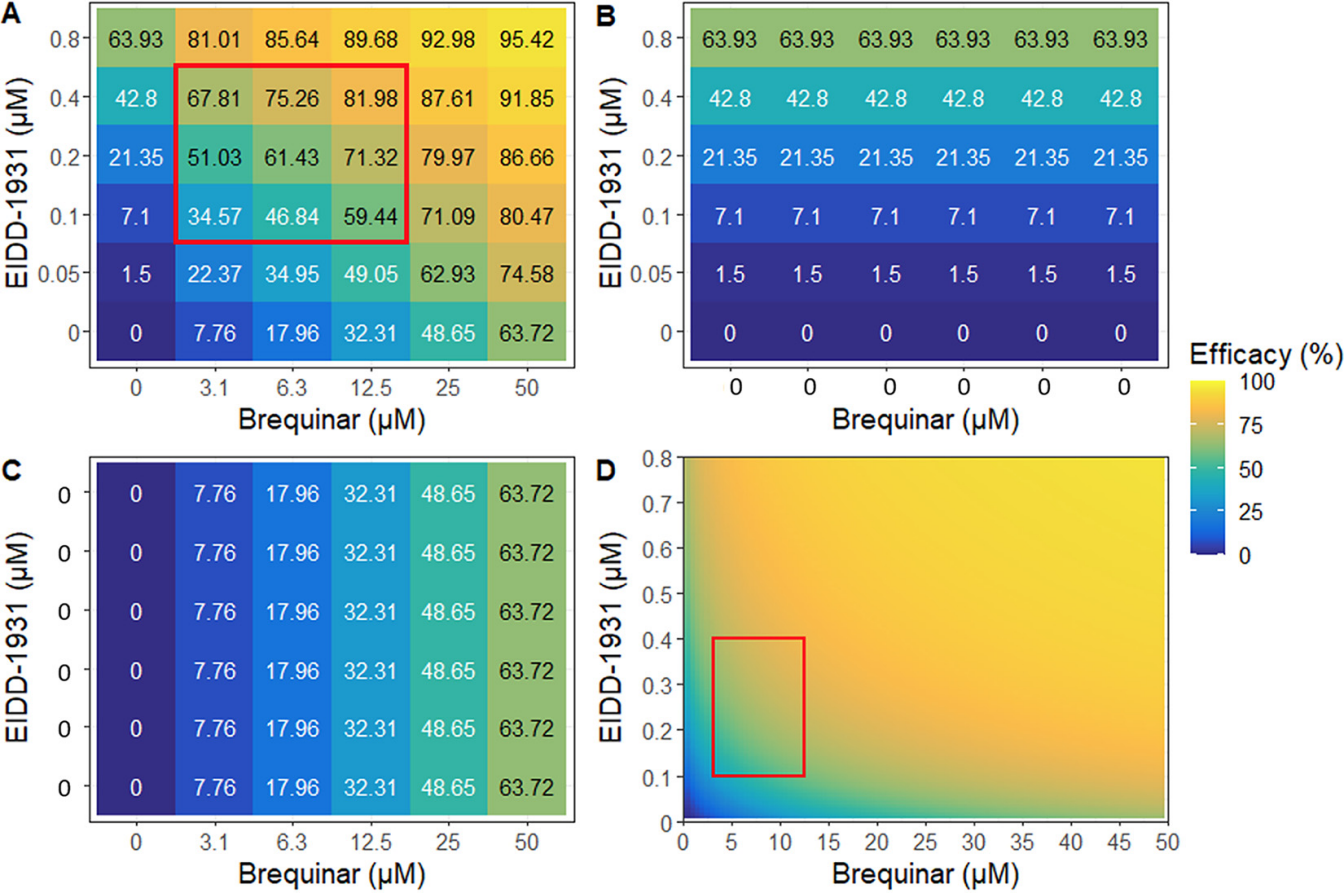
PD modeling of molnupiravir plus brequinar. (A) Model projected efficacy of molnupiravir (EIDD-1931) plus brequinar at empirically tested concentrations. (B) Projected efficacy of EIDD-1931 alone. (C) Projected efficacy of brequinar alone. (D) Heat map of model projected inhibition at all combinatorial concentrations of both agents. The red box denotes the MSA.

For molnupiravir plus brequinar, each drug lacked complete potency when modeled alone even at high concentrations (63.93% inhibition for molnupiravir [Fig. 5B] and 63.72% inhibition for brequinar [Fig. 5C]). However, low concentrations of molnupiravir significantly boosted potency given high concentrations of brequinar (50 μM) (Fig. 5A). This enhanced potency is largely attributable to synergy beyond predicted Bliss independence (multiplicative effects) particularly at low concentrations of molnupiravir (Fig. 5D).

Using model output, we generated heatmaps demonstrating the predicted antiviral potency at all possible combinations of drug concentrations, including those not specifically measured experimentally (Fig. 4D and Fig. 5D; see also Fig. S4D and S5D). The model output can therefore be used to project minute to minute *in vivo* combinatorial antiviral potency of two drugs that have different PK properties of expansion and decay in a person. The data suggest that the combinations may have antiviral potency *in vivo* if observed concentrations can be reached and maintained at the site of infection.

### Activity of a triple drug combination against SARS-CoV-2 in Calu-3 cells.

**FIG 6:**
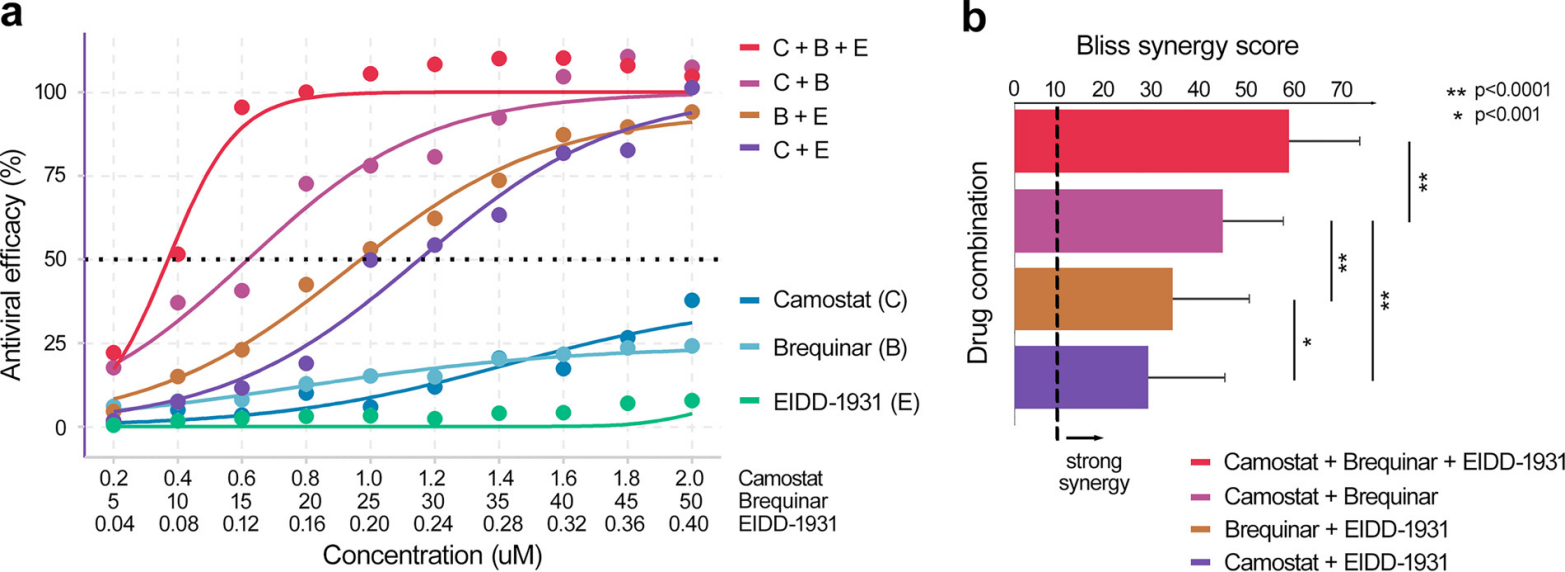
Potent antiviral efficacy with a triple combination of host-targeting (HTA) and viral-targeting (DAA) drugs. (a) Camostat, brequinar, and molnupiravir (EIDD-1931) were mixed at top concentrations of 1.4, 50, and 0.28 μM, respectively, to produce one-, two-, or three-drug combinations. Stock concentrations were serially diluted in 10% increments and added to Calu-3 cells for 2 h prior to infection with the SARS-CoV-2 delta VOC at an MOI 0.01. After 96 h, the cell viability was measured by a CellTiter-Glo assay (Promega), and the antiviral efficacy was calculated as described in Materials and Methods. (b) Bliss synergy scores of the three-drug and two-drug combinations as calculated in SynergyFinder 3.0 (56). The data reflect the averages and standard deviations of triplicate samples per condition, from a single experiment, which was conducted twice with similar results. The dotted vertical line indicates the cutoff for strong Bliss synergy defined previously (23). *P* values are derived from two-sample *t* tests.

Since camostat, brequinar, and molnupiravir inhibit distinct cellular and viral targets and both HTA drugs (camostat and brequinar) synergize with the DAA molnupiravir in pairs, we compared the antiviral efficacy of the triple combination versus the three two-drug combinations against the delta VOC using a recently developed higher-order combination assay (23, 59). The triple combination conferred 100% antiviral efficacy at much lower concentrations of each drug in the cocktail than for any of the three pairwise combinations of the same constituent drugs (Fig. 6a). Moreover, the triple drug combination conferred a significantly higher synergy score than all three two-drug combinations (Fig. 6b). Compared to the two-drug combinations, the triple combination increased both the potency and efficacy of the antiviral effect yet showed no toxic effects on noninfected cells (see Fig. S6).

## DISCUSSION

Besides paxlovid and molnupiravir, there are presently no approved and easy to administer (e.g., oral, intranasal, inhaled) antiviral medications to prevent or ameliorate SARS-CoV-2 infection. Because SARS-CoV-2 continues to evolve rapidly (12–14), drug combination studies such as described in this report provide one step toward more effective drug-based control of the ongoing and possibly future pandemics. Effective drug combinations against HIV and HCV are comprised of two or three DAAs (18, 19). Similarly, we show that combining the two currently approved DAAs, molnupiravir plus paxlovid, confers synergistic suppression of SARS-CoV-2 infection in human lung cells, as we predicted (32), and as recently reported by others (60, 61), including a report that the combination of molnupiravir plus paxlovid appears superior to either drug alone in a mouse model of SARS-CoV-2 (62). We also show that combining HTAs with a DAA provides a similar level of antiviral synergy as an all-DAA combination: three TMPRSS2 inhibitors examined, when combined with molnupiravir, synergistically suppress WT SARS-CoV-2 and the VOC tested. Thus, TMPRSS2 is a potential target for an HTA-containing drug combination, as we have shown here with camostat, nafamostat, and avoralstat. Moreover, investigational inhibitors such as N-0385 (63) and enoxaparin (64) inhibit TMPRSS2 function by distinct mechanisms. In addition to TMPRSS2 cleavage, the SARS-CoV-2 Spike protein is also processed by the cellular enzyme furin. As such, novel furin inhibitors have been shown to synergistically inhibit SARS-CoV-2 infection when combined with camostat (65). Moreover, a phase 2 randomized, double-blind, placebo-controlled clinical trial of oral camostat showed accelerated overall symptom resolution despite no reduction in intranasal viral RNA (66), further supporting the notion that combining other drugs with camostat might enhance clinical and antiviral efficacy. Hence, new TMPRSS2 and furin inhibitors may be worth testing as drug combinations (67, 68).

Brequinar combined with molnupiravir conferred strong antiviral synergy against SARS-CoV-2 infection of Calu-3 cells. Our findings agree with recent studies showing that this HTA+DAA combination is synergistic *in vitro* and conferred superior antiviral effects in mice, although brequinar was administered via the intraperitoneal, not the oral, route in the recent report (31). Brequinar inhibits dihydroorotate dehydrogenase (DHODH), a ubiquitous host enzyme that is required for *de novo* pyrimidine synthesis (69). It was recently shown that brequinar, when combined with dipyridamole, an inhibitor of the pyrimidine salvage pathway (which can be activated when DHODH is inhibited by brequinar), also confers synergistic suppression of SARS-CoV-2 (70). Thus, dipyridamole may provide added benefit to brequinar-containing drug combinations.

We provide a mathematical model that faithfully reproduces experimental data and projects potency against SARS-CoV-2 at combinatorial drug concentrations that were not specifically tested experimentally. This model will serve as the basis for more comprehensive models that include PD data as shown here with human PK for the individual drugs and with SARS-CoV-2 dynamics, to accurately project human *in vivo* potency (71), as we recently did for synergistic drug combinations for Ebola virus (58). Combined PK, PD, and viral dynamics modeling can be conducted for the drug combinations tested here, as well as for others proposed in the literature (32, 72) or revealed in future studies. Lastly, with the triple combination of two HTAs (camostat and brequinar) and a DAA (molnupiravir), we demonstrate the potential of adding a third drug to enhance the potency of a drug pair, echoing the fact that certain drug regimens against HIV and HCV contain three drugs. The next stages of our work will involve further modeling (58) and testing of promising oral drug combinations in mouse models of SARS-CoV-2.

Paxlovid and molnupiravir have 89 and 30% efficacies, respectively, defined as prevention of hospitalization or death (7, 8, 73, 74). While current orally available drugs for SARS-CoV-2 are highly effective at reducing hospitalization, they are not effective for all indications (including postexposure prophylaxis). Although the issue of paxlovid resistance during monotherapy (12–16) has not yet fully emerged clinically, preexisting drug-resistant mutants have been found in therapy naive patients (75, 76). Moreover, resistance to remdesivir has also been observed *in vitro* (77, 78) and clinically (79). Thus, current antivirals are not guaranteed to maintain effectiveness against future variants. We propose that the drug combinations described here may demonstrate increased efficacy *in vivo* and may be of particular importance in immunocompromised hosts who have a difficult time clearing the virus. As investigators are testing combinations of monoclonal antibodies plus a polymerase inhibitor for SARS-CoV-2 (80) and small molecule drug combinations for influenza (27), we advocate for further modeling and *in vivo* testing of oral small molecule drug combinations containing both HTAs and DAAs versus SARS-CoV-2, VOC, other coronaviruses, and other viruses for pandemic response and preparation (32).

## MATERIALS AND METHODS

### Chemicals, cell culture, and live virus.

Vero E6 and Calu-3 cells were maintained in standard medium (minimum essential medium; Gibco, catalog no. 11095) supplemented with 9% fetal bovine serum (FBS; HyClone, SH3007103) and 1% penicillin-streptomycin (Gibco, catalog no. 15140); HEK293T cells expressing human ACE2 and TMPRSS2 (55) (here called 293TAT cells), provided by Carol Weiss, were maintained in Dulbecco modified Eagle medium (DMEM; Gibco, catalog no. 11995), 9% FBS (HyClone, SH3007103), 1% penicillin-streptomycin (Gibco, catalog no. 15140), 1% nonessential amino acids (Gibco, catalog no. 11140), and 20 mM HEPES (Gibco, catalog no. 15630). HTA and DAA compounds were purchased from commercial vendors as outlined in Table S1 in the supplemental material.

Wild-type, infectious SARS-CoV-2 was obtained from BEI Resources (isolate USA-WA1/2020 NR-52281). The SARS-CoV_2 VOC stocks of beta and delta viruses were generated as follows. The beta virus (B.1.351 lineage), isolate hCoV-19/South Africa/KRISP-K005325/2020 (NR-54009; GISAID: EPI_ISL_678615) was originally obtained from BEI Resources, NIAID, NIH, and further expanded in Vero-TMPRSS2 cells in DMEM containing 2% heat-inactivated FBS (HI-FBS). The delta virus (GISAID accession number EPI_ISL_13858636) was isolated from local Seattle nasopharyngeal swab samples of a SARS-CoV-2-infected patient stored in viral transport medium, kindly provided by Alex Greninger. Briefly, the viruses in the VTM were first expanded using Vero-ACE2-TMPRSS2 cells in DMEM containing 2% HI-FBS. The initial crude expansion was then plaque-purified in Vero-ACE2-TMPRSS2 cells. The plaque-purified viruses were sent for amplicon sequencing (Swift Biosciences) to confirm their genome sequence. Virus was further amplified in Vero-TMPRSS2 cells. Virus titers were measured by plaque assays on Vero-TMPRSS2 cells.

### Drug treatment and infection of cells with live virus.

Compounds were added to cell plates and 2 h later, live virus (SARS-CoV-2 WA1 at a multiplicity of infection [MOI] of 0.1 or delta or beta VOC at MOIs of 0.01) was added to each infected well, or virus-free medium was added to each uninfected well. Plates were incubated at 37°C and 5% CO_2_ for 48 or 96 h 293TAT cells or Calu-3 cells, respectively. The assay readout was the cell viability based on a CellTiter-Glo assay (Promega).

### Cell viability assay.

For drug dose-response studies, we measured cell viability in parallel wells using the CellTiter-Glo 2.0 assay (Promega). The assay measures the number of viable cells in culture by quantifying ATP, which indicates the presence of metabolically active cells. The contents of each well were aspirated, followed by the addition of 50 μL of phosphate-buffered saline (Gibco, catalog no. 10010031) and 50 μL of CellTiter-Glo reagent. The plate was then shaken for 2 min, incubated at room temperature for 10 min, and read for luminescence on a BioTek Synergy H4 plate reader.

### Drug combination assays.

Checkerboard assays of two drug combinations were performed as described earlier (23). Briefly, two drugs were tested in dose responses consisting of dimethyl sulfoxide (DMSO) control and five concentrations of each drug, yielding six concentrations of drug A and six concentrations of drug B. Combining the two separate drug dose responses creates a matrix or checkerboard of 36 dose-combinations of the two drugs. Calu-3 (*n* = 50,000) or 293TAT (*n* = 10,000) cells were seeded into each well of 96-well black, clear-bottom plates in the cell line’s respective medium. Each two-drug concentration tested in a 6 × 6 checkerboard assay was performed in duplicate for 293TAT cells or triplicate for Calu-3 cells. Cells were infected, and parallel wells were not infected to assess the toxicity of the drugs. Both plates were incubated at 37°C and 5% CO_2_ for 48 h (for 293TAT cells) or 96 h (for Calu-3 cells) prior to the CTG assay. For three drug combination studies, we employed a method that samples the diagonal space of the three-dimensional checkerboard (23, 59). Briefly, camostat, brequinar, and molnupiravir (EIDD-1931) were mixed at top concentrations of 1.4, 50, and 0.28 μM, respectively, to produce one-, two-, and three-drug combinations. Stock concentrations were serially diluted in 10% increments and added to Calu-3 cells at 2 h prior to infection. After 96 h, cell viability was measured by a CellTiter-Glo assay (Promega).

### Data analyses.

For single-drug experiments, drug concentrations were log transformed, and the concentrations of drug(s) that inhibited virus by 50% (i.e., IC_50_) and the concentrations of drug(s) that killed 50% of cells (i.e., CC_50_) were determined via nonlinear logistic regressions of log(inhibitor) versus response-variable dose-response functions (four parameters) constrained to zero bottom asymptote by statistical analysis using Prism 9 (GraphPad Software, Inc.), as described previously (23). Input data for these equations consisted of relative light units (RLU) generated by CellTiter-Glo assays. The selectivity index was calculated by dividing the CC_50_ by the IC_50_. The antiviral efficacy was calculated by comparing the RLU from virus-infected (IFX) cells treated with drug to the average of the infected cells treated with solvent (DMSO) and expressed as a percentage relative to the virus-induced cytopathic effect: RLU of noninfected/DMSO-treated cells – RLU in infected DMSO treated cells, using the following equation:

$$\left(\frac{\left(\text{IFX/drug }-\text{ average IFX/DMSO}\right)}{\left(\text{average}\;\text{non}-\text{IFX/DMSO}-\text{average IFX/DMSO}\right)}\right)\times 100$$

Cell viability was calculated by comparing the RLU from noninfected cells treated with drugs to the noninfected cells treated with DMSO:

$$\left(\frac{\text{non}-\text{IFX/drug}}{\text{average}\;\text{non}-\text{IFX/DMSO}}\right)\times 100$$

Dose-response data from checkerboard assays were analyzed in SynergyFinder3, an open-access platform for multidrug combination synergies (56). Several combination parameters were reported from SynergyFinder3, including the average Bliss Synergy Score of the entire dose-response matrix and the maximum synergistic area (MSA), which corresponds to the maximum Bliss score calculated over an area of nine doses of the two compounds in a checkerboard experiment (i.e., 3 × 3 dose-response submatrix). Synergy is defined as when the observed inhibition is greater than that predicted by multiplicative Bliss independence at a given set of drug concentrations. For triple-drug experiments, the three-drug combination was compared to the three two-drug combinations of the drugs that comprise the triple combination using SynergyFinder3.0. Finally, SynToxProfiler (57) was used to evaluate the toxicity profile of the drug combinations in noninfected cells via checkerboard assays. For this, toxicity was calculated by subtracting the viability results above from 100 (to convert to percent inhibition values), and checkerboard toxicity data were uploaded to SynToxProfiler (see Fig. S2).

### Mathematical modeling.

We previously modified the Bliss independence PD model (81) to directly assess the potency of drug combinations across different drug concentrations. The purpose of the model was to recapitulate observed experimental data and then make projections about combinatorial drug potency at concentrations that were not measured experimentally. The model can then be synchronized with PK models to predict the percentage of new cell infections being prevented at any point during the drug dosing interval. The model includes the parameters IC_50_ (the drug concentration at which 50% of infections are prevented), Hill coefficients of both drugs (*h*_1_ and *h*_2_, the slope of the dose-response curve), and an exponent (*a*) for the assessment of synergy (58), as expressed in the following equation:

$${\text{Efficacy}}_{\text{combo}}=100\times {\left(\frac{1}{1+{\left(\frac{D_{1}}{IC_{50,1}}\right)}^{h1}}\times \frac{1}{1+{\left(\frac{D_{2}}{IC_{50,2}}\right)}^{h2}}\right)}^{a}$$

## Supplementary Material

Reviewer comments

## References

[1] Weinreich DM, Sivapalasingam S, Norton T, Ali S, Gao H, Bhore R, Musser BJ, Soo Y, Rofail D, Im J, Perry C, Pan C, Hosain R, Mahmood A, Davis JD, Turner KC, Hooper AT, Hamilton JD, Baum A, Kyratsous CA, Kim Y, Cook A, Kampman W, Kohli A, Sachdeva Y, Graber X, Kowal B, DiCioccio T, Stahl N, Lipsich L, Braunstein N, Herman G, Yancopoulos GD, Trial Investigators. 2021. REGN-COV2, a neutralizing antibody cocktail, in outpatients with Covid-19. N Engl J Med 384:238–251. doi:10.1056/NEJMoa2035002. 33332778 PMC7781102

[2] O’Brien MP, Forleo-Neto E, Musser BJ, Isa F, Chan K-C, Sarkar N, Bar KJ, Barnabas RV, Barouch DH, Cohen MS, Hurt CB, Burwen DR, Marovich MA, Hou P, Heirman I, Davis JD, Turner KC, Ramesh D, Mahmood A, Hooper AT, Hamilton JD, Kim Y, Purcell LA, Baum A, Kyratsous CA, Krainson J, Perez-Perez R, Mohseni R, Kowal B, DiCioccio AT, Stahl N, Lipsich L, Braunstein N, Herman G, Yancopoulos GD, Weinreich DM, Covid-19 Phase 3 Prevention Trial Team. 2021. Subcutaneous REGEN-COV antibody combination to prevent Covid-19. N Engl J Med 385:1184–1195. doi:10.1056/NEJMoa2109682. 34347950 PMC8362593

[3] Hoffmann M, Kruger N, Schulz S, Cossmann A, Rocha C, Kempf A, Nehlmeier I, Graichen L, Moldenhauer AS, Winkler MS, Lier M, Dopfer-Jablonka A, Jack HM, Behrens GMN, Pohlmann S. 2022. The Omicron variant is highly resistant against antibody-mediated neutralization: implications for control of the COVID-19 pandemic. Cell 185:447–456 e11. doi:10.1016/j.cell.2021.12.032. 35026151 PMC8702401

[4] Gottlieb RL, Vaca CE, Paredes R, Mera J, Webb BJ, Perez G, Oguchi G, Ryan P, Nielsen BU, Brown M, Hidalgo A, Sachdeva Y, Mittal S, Osiyemi O, Skarbinski J, Juneja K, Hyland RH, Osinusi A, Chen S, Camus G, Abdelghany M, Davies S, Behenna-Renton N, Duff F, Marty FM, Katz MJ, Ginde AA, Brown SM, Schiffer JT, Hill JA, GS-US-540-9012 (PINETREE) Investigators. 2022. Early remdesivir to prevent progression to severe Covid-19 in outpatients. N Engl J Med 386:305–315. doi:10.1056/NEJMoa2116846. 34937145 PMC8757570

[5] Ader F, Bouscambert-Duchamp M, Hites M, Peiffer-Smadja N, Poissy J, Belhadi D, Diallo A, Le MP, Peytavin G, Staub T, Greil R, Guedj J, Paiva JA, Costagliola D, Yazdanpanah Y, Burdet C, Mentre F, DisCoVeRy Study Group. 2022. Remdesivir plus standard of care versus standard of care alone for the treatment of patients admitted to hospital with COVID-19 (DisCoVeRy): a phase 3, randomised, controlled, open-label trial. Lancet Infect Dis 22:209–221. doi:10.1016/S1473-3099(21)00485-0. 34534511 PMC8439621

[6] Beigel JH, Tomashek KM, Dodd LE, Mehta AK, Zingman BS, Kalil AC, Hohmann E, Chu HY, Luetkemeyer A, Kline S, Lopez de Castilla D, Finberg RW, Dierberg K, Tapson V, Hsieh L, Patterson TF, Paredes R, Sweeney DA, Short WR, Touloumi G, Lye DC, Ohmagari N, Oh MD, Ruiz-Palacios GM, Benfield T, Fatkenheuer G, Kortepeter MG, Atmar RL, Creech CB, Lundgren J, Babiker AG, Pett S, Neaton JD, Burgess TH, Bonnett T, Green M, Makowski M, Osinusi A, Nayak S, Lane HC, ACTT-1 Study Group Members. 2020. Remdesivir for the treatment of Covid-19: preliminary report. N Engl J Med 383:1813–1826. doi:10.1056/NEJMoa2007764. 32445440 PMC7262788

[7] Fischer WA, II, Eron JJ, Jr, Holman W, Cohen MS, Fang L, Szewczyk LJ, Sheahan TP, Baric R, Mollan KR, Wolfe CR, Duke ER, Azizad MM, Borroto-Esoda K, Wohl DA, Coombs RW, James Loftis A, Alabanza P, Lipansky F, Painter WP. 2022. A phase 2a clinical trial of molnupiravir in patients with COVID-19 shows accelerated SARS-CoV-2 RNA clearance and elimination of infectious virus. Sci Transl Med 14:eabl7430. doi:10.1126/scitranslmed.abl7430. 34941423 PMC10763622

[8] Hammond J, Leister-Tebbe H, Gardner A, Abreu P, Bao W, Wisemandle W, Baniecki M, Hendrick VM, Damle B, Simon-Campos A, Pypstra R, Rusnak JM, EPIC-HR Investigators. 2022. Oral nirmatrelvir for high-risk, nonhospitalized adults with Covid-19. N Engl J Med 386:1397–1408. doi:10.1056/NEJMoa2118542. 35172054 PMC8908851

[9] Lemaitre F, Gregoire M, Monchaud C, Bouchet S, Saint-Salvi B, Polard E, SFPT Therapeutic Drug Monitoring and Treatment Personalization Group (STP-PT) of the French Society of Pharmacology and Therapeutics, French Pharmacovigilance Network, ANRS-MIE AC-43 Clinical Pharmacology Committee Joint Working Group, Monitoring STD, SFPT STP-PT, Therapeutics, French Pharmacovigilance Network, ANRS-MIE AC-43 Clinical Pharmacology Committee Joint Working Group. 2022. Management of drug-drug interactions with nirmatrelvir/ritonavir in patients treated for Covid-19: guidelines from the French Society of Pharmacology and Therapeutics (SFPT). Therapie doi:10.1016/j.therap.2022.03.005. PMC9020499 35618549

[10] Carlin AF, Clark AE, Chaillon A, Garretson AF, et al. 2022. Case report: virologic and immunologic characterization of COVID-19 recrudescence after nirmatrelvir/ritonavir treatment. 10.21203/rs.3.rs-1662783/v1. Accessed 24 May 2022.

[11] Rubin R. 2022. From positive to negative to positive again: the mystery of why COVID-19 rebounds in some patients who take Paxlovid. JAMA 327:2380–2382. doi:10.1001/jama.2022.9925. 35675094

[12] Jochmans D, Liu C, Donckers K, Stoycheva A, Boland S, Stevens SK, De Vita C, Vanmechelen B, Maes P, Trüeb B, Ebert N, Thiel V, De Jonghe S, Vangeel L, Bardiot D, Jekle A, Blatt LM, Beigelman L, Symons JA, Raboisson P, Chaltin P, Marchand A, Neyts J, Deval J, Vandyck K. 2022. The substitutions L50F, E166A and L167F in SARS-CoV-2 3CLpro are selected by a protease inhibitor *in vitro* and confer resistance to nirmatrelvir. bioRxiv. https://www.biorxiv.org/content/10.1101/2022.06.07.495116v1. 10.1128/mbio.02815-22 PMC9973015 36625640

[13] Zhou Y, Gammeltoft KA, Ryberg LA, Pham LV, Fahnøe U, Binderup A, Hernandez CRD, Offersgaard A, Fernandez-Antunez C, Peters GHJ, Ramirez S, Bukh J, Gottwein JM. 2022. Nirmatrelvir-resistant SARS-CoV-2 variants with high fitness *in vitro*. bioRxiv. https://www.biorxiv.org/content/10.1101/2022.06.06.494921v1. 10.1126/sciadv.add7197 PMC9770952 36542720

[14] Flynn JM, Samant N, Schneider-Nachum G, Barkan DT, Yilmaz NK, Schiffer CA, Moquin SA, Dovala D, Bolon DNA. 2022. Comprehensive fitness landscape of SARS-CoV-2 M(pro) reveals insights into viral resistance mechanisms. Elife 11. doi:10.7554/eLife.77433. PMC9323007 35723575

[15] Heilmann E, Costacurta F, Volland A, von Laer D. 2022. SARS-CoV-2 3CLpro mutations confer resistance to paxlovid (nirmatrelvir/ritonavir) in a VSV-based, non-gain-of-function system. bioRxiv. https://www.biorxiv.org/content/10.1101/2022.07.02.495455v1.

[16] Iketani S, Mohri H, Culbertson B, Hong SJ, Duan Y, Luck MI, Annavajhala MK, Guo Y, Sheng Z, Uhlemann A-C, Goff SP, Sabo Y, Yang H, Chavez A, Ho DD. 2022. Multiple pathways for SARS-CoV-2 resistance to nirmatrelvir. bioRxiv. https://www.biorxiv.org/content/10.1101/2022.08.07.499047v2. 10.1038/s41586-022-05514-2 PMC9849135 36351451

[17] Schiffer JT, Johnston C, Wald A, Corey L. 2020. An early test-and-treat strategy for severe acute respiratory syndrome coronavirus 2. Open Forum Infect Dis 7:ofaa232. doi:10.1093/ofid/ofaa232. 32661497 PMC7313828

[18] Cihlar T, Fordyce M. 2016. Current status and prospects of HIV treatment. Curr Opin Virol 18:50–56. doi:10.1016/j.coviro.2016.03.004. 27023283

[19] Sarrazin C. 2021. Treatment failure with DAA therapy: importance of resistance. J Hepatol 74:1472–1482. doi:10.1016/j.jhep.2021.03.004. 33716089

[20] Dyall J, Nelson EA, DeWald LE, Guha R, Hart BJ, Zhou H, Postnikova E, Logue J, Vargas WM, Gross R, Michelotti J, Deiuliis N, Bennett RS, Crozier I, Holbrook MR, Morris PJ, Klumpp-Thomas C, McKnight C, Mierzwa T, Shinn P, Glass PJ, Johansen LM, Jahrling PB, Hensley LE, Olinger GG, Jr, Thomas C, White JM. 2018. Identification of combinations of approved drugs with synergistic activity against Ebola virus in cell cultures. J Infect Dis 218:S672–S678. doi:10.1093/infdis/jiy304. 29939303 PMC6249579

[21] Sun W, He S, Martinez-Romero C, Kouznetsova J, Tawa G, Xu M, Shinn P, Fisher E, Long Y, Motabar O, Yang S, Sanderson PE, Williamson PR, Garcia-Sastre A, Qiu X, Zheng W. 2017. Synergistic drug combination effectively blocks Ebola virus infection. Antiviral Res 137:165–172. doi:10.1016/j.antiviral.2016.11.017. 27890675 PMC5182099

[22] Bekerman E, Neveu G, Shulla A, Brannan J, Pu SY, Wang S, Xiao F, Barouch-Bentov R, Bakken RR, Mateo R, Govero J, Nagamine CM, Diamond MS, De Jonghe S, Herdewijn P, Dye JM, Randall G, Einav S. 2017. Anticancer kinase inhibitors impair intracellular viral trafficking and exert broad-spectrum antiviral effects. J Clin Invest 127:1338–1352. doi:10.1172/JCI89857. 28240606 PMC5373883

[23] Herring S, Oda JM, Wagoner J, Kirchmeier D, O’Connor A, Nelson EA, Huang Q, Liang Y, DeWald LE, Johansen LM, Glass PJ, Olinger GG, Ianevski A, Aittokallio T, Paine MF, Fink SL, White JM, Polyak SJ. 2021. Inhibition of arenaviruses by combinations of orally available approved drugs. Antimicrob Agents Chemother 65:e01146-20. doi:10.1128/AAC.01146-20. PMC8097473 33468464

[24] Nguyen JT, Hoopes JD, Smee DF, Prichard MN, Driebe EM, Engelthaler DM, Le MH, Keim PS, Spence RP, Went GT. 2009. Triple combination of oseltamivir, amantadine, and ribavirin displays synergistic activity against multiple influenza virus strains *in vitro*. Antimicrob Agents Chemother 53:4115–4126. doi:10.1128/AAC.00476-09. 19620324 PMC2764153

[25] Nguyen JT, Hoopes JD, Le MH, Smee DF, Patick AK, Faix DJ, Blair PJ, de Jong MD, Prichard MN, Went GT. 2010. Triple combination of amantadine, ribavirin, and oseltamivir is highly active and synergistic against drug resistant influenza virus strains *in vitro*. PLoS One 5:e9332. doi:10.1371/journal.pone.0009332. 20179772 PMC2825274

[26] Ashtiwi NM, Sarr D, Nagy T, Reneer ZB, Tripp RA, Rada B. 2022. The hypothiocyanite and amantadine combination treatment prevents lethal influenza A virus infection in mice. Front Immunol 13:859033. doi:10.3389/fimmu.2022.859033. 35663985 PMC9159274

[27] Koszalka P, George A, Dhanasekaran V, Hurt AC, Subbarao K. 2022. Effect of baloxavir and oseltamivir in combination on infection with influenza viruses with PA/I38T or PA/E23K substitutions in the ferret model. mBio 13:e01056-22. doi:10.1128/mbio.01056-22. 35938724 PMC9426601

[28] Bobrowski T, Chen L, Eastman RT, Itkin Z, Shinn P, Chen CZ, Guo H, Zheng W, Michael S, Simeonov A, Hall MD, Zakharov AV, Muratov EN. 2021. Synergistic and antagonistic drug combinations against SARS-CoV-2. Mol Ther 29:873–885. doi:10.1016/j.ymthe.2020.12.016. 33333292 PMC7834738

[29] Ianevski A, Yao R, Biza S, Zusinaite E, Mannik A, Kivi G, Planken A, Kurg K, Tombak E-M, Ustav M, Shtaida N, Kulesskiy E, Jo E, Yang J, Lysvand H, Løseth K, Oksenych V, Aas PA, Tenson T, Vitkauskienė A, Windisch MP, Fenstad MH, Nordbø SA, Ustav M, Bjørås M, Kainov DE. 2020. Identification and tracking of antiviral drug combinations. Viruses 12:1178. doi:10.3390/v12101178. 33080984 PMC7589631

[30] Ianevski A, Yao R, Lysvand H, Grodeland G, Legrand N, Oksenych V, Zusinaite E, Tenson T, Bjoras M, Kainov DE. 2021. Nafamostat-interferon-alpha combination suppresses SARS-CoV-2 infection *in vitro* and *in vivo* by cooperatively targeting host TMPRSS2. Viruses 13:1768. doi:10.3390/v13091768. 34578348 PMC8473362

[31] Schultz DC, Johnson RM, Ayyanathan K, Miller J, Whig K, Kamalia B, Dittmar M, Weston S, Hammond HL, Dillen C, Ardanuy J, Taylor L, Lee JS, Li M, Lee E, Shoffler C, Petucci C, Constant S, Ferrer M, Thaiss CA, Frieman MB, Cherry S. 2022. Pyrimidine inhibitors synergize with nucleoside analogues to block SARS-CoV-2. Nature 604:134–140. doi:10.1038/s41586-022-04482-x. 35130559 PMC10377386

[32] White JM, Schiffer JT, Bender Ignacio RA, Xu S, Kainov D, Ianevski A, Aittokallio T, Frieman M, Olinger GG, Polyak SJ. 2021. Drug combinations as a first line of defense against coronaviruses and other emerging viruses. mBio 12:e03347-21. doi:10.1128/mbio.03347-21. 34933447 PMC8689562

[33] Owen DR, Allerton CMN, Anderson AS, Aschenbrenner L, Avery M, Berritt S, Boras B, Cardin RD, Carlo A, Coffman KJ, Dantonio A, Di L, Eng H, Ferre R, Gajiwala KS, Gibson SA, Greasley SE, Hurst BL, Kadar EP, Kalgutkar AS, Lee JC, Lee J, Liu W, Mason SW, Noell S, Novak JJ, Obach RS, Ogilvie K, Patel NC, Pettersson M, Rai DK, Reese MR, Sammons MF, Sathish JG, Singh RSP, Steppan CM, Stewart AE, Tuttle JB, Updyke L, Verhoest PR, Wei L, Yang Q, Zhu Y. 2021. An oral SARS-CoV-2 M(pro) inhibitor clinical candidate for the treatment of COVID-19. Science 374:1586–1593. doi:10.1126/science.abl4784. 34726479

[34] Sonnleitner ST, Prelog M, Sonnleitner S, Hinterbichler E, Halbfurter H, Kopecky DBC, Almanzar G, Koblmuller S, Sturmbauer C, Feist L, Horres R, Posch W, Walder G. 2022. Cumulative SARS-CoV-2 mutations and corresponding changes in immunity in an immunocompromised patient indicate viral evolution within the host. Nat Commun 13:2560. doi:10.1038/s41467-022-30163-4. 35538074 PMC9090742

[35] Weigang S, Fuchs J, Zimmer G, Schnepf D, Kern L, Beer J, Luxenburger H, Ankerhold J, Falcone V, Kemming J, Hofmann M, Thimme R, Neumann-Haefelin C, Ulferts S, Grosse R, Hornuss D, Tanriver Y, Rieg S, Wagner D, Huzly D, Schwemmle M, Panning M, Kochs G. 2021. Within-host evolution of SARS-CoV-2 in an immunosuppressed COVID-19 patient as a source of immune escape variants. Nat Commun 12:6405. doi:10.1038/s41467-021-26602-3. 34737266 PMC8568958

[36] Voloch CM, da Silva Francisco R, Jr, de Almeida LGP, Brustolini OJ, Cardoso CC, Gerber AL, Guimaraes APC, Leitao IC, Mariani D, Ota VA, Lima CX, Teixeira MM, Dias ACF, Galliez RM, Faffe DS, Porto LC, Aguiar RS, Castineira T, Ferreira OC, Tanuri A, de Vasconcelos ATR. 2021. Intra-host evolution during SARS-CoV-2 prolonged infection. Virus Evol 7:veab078. doi:10.1093/ve/veab078. 34642605 PMC8500031

[37] Corey L, Beyrer C, Cohen MS, Michael NL, Bedford T, Rolland M. 2021. SARS-CoV-2 variants in patients with immunosuppression. N Engl J Med 385:562–566. doi:10.1056/NEJMsb2104756. 34347959 PMC8494465

[38] Ison MG, Portsmouth S, Yoshida Y, Shishido T, Mitchener M, Tsuchiya K, Uehara T, Hayden FG. 2020. Early treatment with baloxavir marboxil in high-risk adolescent and adult outpatients with uncomplicated influenza (CAPSTONE-2): a randomised, placebo-controlled, phase 3 trial. Lancet Infect Dis 20:1204–1214. doi:10.1016/S1473-3099(20)30004-9. 32526195

[39] Mulangu S, Dodd LE, Davey RT, Tshiani Mbaya O, Proschan M, Mukadi D, Lusakibanza Manzo M, Nzolo D, Tshomba Oloma A, Ibanda A, Ali R, Coulibaly S, Levine AC, Grais R, Diaz J, Lane HC, Muyembe-Tamfum J-J, Sivahera B, Camara M, Kojan R, Walker R, Dighero-Kemp B, Cao H, Mukumbayi P, Mbala-Kingebeni P, Ahuka S, Albert S, Bonnett T, Crozier I, Duvenhage M, PALM Consortium Study Team, et al. 2019. A randomized, controlled trial of Ebola virus disease therapeutics. N Engl J Med 381:2293–2303. doi:10.1056/NEJMoa1910993. 31774950 PMC10680050

[40] Group ISS, Lundgren JD, Babiker AG, Gordin F, Emery S, Grund B, Sharma S, Avihingsanon A, Cooper DA, Fatkenheuer G, Llibre JM, Molina JM, Munderi P, Schechter M, Wood R, Klingman KL, Collins S, Lane HC, Phillips AN, Neaton JD. 2015. Initiation of antiretroviral therapy in early asymptomatic HIV infection. N Engl J Med 373:795–807. 26192873 10.1056/NEJMoa1506816 PMC4569751

[41] Wood MJ, Shukla S, Fiddian AP, Crooks RJ. 1998. Treatment of acute herpes zoster: effect of early (< 48 h) versus late (48–72 h) therapy with acyclovir and valaciclovir on prolonged pain. J Infect Dis 178(Suppl 1):S81–S84. doi:10.1086/514271. 9852981

[42] Hoffmann M, Kleine-Weber H, Schroeder S, Kruger N, Herrler T, Erichsen S, Schiergens TS, Herrler G, Wu NH, Nitsche A, Muller MA, Drosten C, Pohlmann S. 2020. SARS-CoV-2 cell entry depends on ACE2 and TMPRSS2 and is blocked by a clinically proven protease inhibitor. Cell 181:271–280. doi:10.1016/j.cell.2020.02.052. 32142651 PMC7102627

[43] Sheahan TP, Sims AC, Zhou S, Graham RL, Pruijssers AJ, Agostini ML, Leist SR, Schafer A, Dinnon KH, III, Stevens LJ, Chappell JD, Lu X, Hughes TM, George AS, Hill CS, Montgomery SA, Brown AJ, Bluemling GR, Natchus MG, Saindane M, Kolykhalov AA, Painter G, Harcourt J, Tamin A, Thornburg NJ, Swanstrom R, Denison MR, Baric RS. 2020. An orally bioavailable broad-spectrum antiviral inhibits SARS-CoV-2 in human airway epithelial cell cultures and multiple coronaviruses in mice. Sci Transl Med 12:eabb5883. doi:10.1126/scitranslmed.abb5883. 32253226 PMC7164393

[44] Hoffmann M, Schroeder S, Kleine-Weber H, Muller MA, Drosten C, Pohlmann S. 2020. Nafamostat mesylate blocks activation of SARS-CoV-2: new treatment option for COVID-19. Antimicrob Agents Chemother 64:e00754-20. doi:10.1128/AAC.00754-20. 32312781 PMC7269515

[45] Sun YJ, Velez G, Parsons DE, Li K, Ortiz ME, Sharma S, McCray PB, Jr, Bassuk AG, Mahajan VB. 2021. Structure-based phylogeny identifies avoralstat as a TMPRSS2 inhibitor that prevents SARS-CoV-2 infection in mice. J Clin Invest 131. 10.1172/JCI147973 PMC8121520 33844653

[46] Li K, Meyerholz DK, Bartlett JA, McCray PB, Jr. 2021. The TMPRSS2 inhibitor nafamostat reduces SARS-CoV-2 pulmonary infection in mouse models of COVID-19. mBio 12:e00970-21. doi:10.1128/mBio.00970-21. 34340553 PMC8406266

[47] Gunst JD, Staerke NB, Pahus MH, Kristensen LH, Bodilsen J, Lohse N, Dalgaard LS, Brønnum D, Fröbert O, Hønge B, Johansen IS, Monrad I, Erikstrup C, Rosendal R, Vilstrup E, Mariager T, Bove DG, Offersen R, Shakar S, Cajander S, Jørgensen NP, Sritharan SS, Breining P, Jespersen S, Mortensen KL, Jensen ML, Kolte L, Frattari GS, Larsen CS, Storgaard M, Nielsen LP, Tolstrup M, Sædder EA, Østergaard LJ, Ngo HT, Jensen MH, Højen JF, Kjolby M, Søgaard OS. 2021. Efficacy of the TMPRSS2 inhibitor camostat mesilate in patients hospitalized with Covid-19: a double-blind randomized controlled trial. EClinicalMedicine 35:100849. doi:10.1016/j.eclinm.2021.100849. 33903855 PMC8060682

[48] Zhuravel SV, Khmelnitskiy OK, Burlaka OO, Gritsan AI, Goloshchekin BM, Kim S, Hong KY. 2021. Nafamostat in hospitalized patients with moderate to severe COVID-19 pneumonia: a randomised phase II clinical trial. EClinicalMedicine 41:101169. doi:10.1016/j.eclinm.2021.101169. 34723164 PMC8548051

[49] Quinn TM, Gaughan EE, Bruce A, Antonelli J, O’Connor R, Li F, McNamara S, Koch O, MacKintosh C, Dockrell D, Walsh T, Blyth KG, Church C, Schwarze J, Boz C, Valanciute A, Burgess M, Emanuel P, Mills B, Rinaldi G, Hardisty G, Mills R, Findlay EG, Jabbal S, Duncan A, Plant S, Marshall ADL, Young I, Russell K, Scholefield E, Nimmo AF, Nazarov IB, Churchill GC, McCullagh JSO, Ebrahimi KH, Ferrett C, Templeton K, Rannard S, Owen A, Moore A, Finlayson K, Shankar-Hari M, Norrie J, Parker RA, Akram AR, Anthony DC, Dear JW, Hirani N, Dhaliwal K. 2022. Randomised controlled trial of intravenous nafamostat mesylate in COVID pneumonitis: phase 1b/2a experimental study to investigate safety, pharmacokinetics and pharmacodynamics. EBioMedicine 76:103856. doi:10.1016/j.ebiom.2022.103856. 35152152 PMC8831100

[50] Han Y, Duan X, Yang L, Nilsson-Payant BE, Wang P, Duan F, Tang X, Yaron TM, Zhang T, Uhl S, Bram Y, Richardson C, Zhu J, Zhao Z, Redmond D, Houghton S, Nguyen DT, Xu D, Wang X, Jessurun J, Borczuk A, Huang Y, Johnson JL, Liu Y, Xiang J, Wang H, Cantley LC, tenOever BR, Ho DD, Pan FC, Evans T, Chen HJ, Schwartz RE, Chen S. 2021. Identification of SARS-CoV-2 inhibitors using lung and colonic organoids. Nature 589:270–275. doi:10.1038/s41586-020-2901-9. 33116299 PMC8034380

[51] Riva L, Yuan S, Yin X, Martin-Sancho L, Matsunaga N, Pache L, Burgstaller-Muehlbacher S, De Jesus PD, Teriete P, Hull MV, Chang MW, Chan JF, Cao J, Poon VK, Herbert KM, Cheng K, Nguyen TH, Rubanov A, Pu Y, Nguyen C, Choi A, Rathnasinghe R, Schotsaert M, Miorin L, Dejosez M, Zwaka TP, Sit KY, Martinez-Sobrido L, Liu WC, White KM, Chapman ME, Lendy EK, Glynne RJ, Albrecht R, Ruppin E, Mesecar AD, Johnson JR, Benner C, Sun R, Schultz PG, Su AI, Garcia-Sastre A, Chatterjee AK, Yuen KY, Chanda SK. 2020. Discovery of SARS-CoV-2 antiviral drugs through large-scale compound repurposing. Nature 586:113–119. doi:10.1038/s41586-020-2577-1. 32707573 PMC7603405

[52] Leneva I, Kartashova N, Poromov A, Gracheva A, Korchevaya E, Glubokova E, Borisova O, Shtro A, Loginova S, Shchukina V, Khamitov R, Faizuloev E. 2021. Antiviral activity of umifenovir *in vitro* against a broad spectrum of coronaviruses, including the novel SARS-CoV-2 virus. Viruses 13:1665. doi:10.3390/v13081665. 34452529 PMC8402645

[53] Wang X, Cao R, Zhang H, Liu J, Xu M, Hu H, Li Y, Zhao L, Li W, Sun X, Yang X, Shi Z, Deng F, Hu Z, Zhong W, Wang M. 2020. The anti-influenza virus drug, arbidol is an efficient inhibitor of SARS-CoV-2 *in vitro*. Cell Discov 6:28. doi:10.1038/s41421-020-0169-8. 32373347 PMC7195821

[54] Ianevski A, Yao R, Simonsen RM, Myhre V, Ravlo E, Kaynova GD, Zusinaite E, White JM, Pan Q, Polyak SJ, Oksenych V, Windisch MP, Lastauskiene E, Vitkauskiene A, Matukevicius A, Tenson T, Bjoras M, Kainov DE. 2022. Broad-spectrum mono- and combinational drug therapies for global viral pandemic preparedness. bioRxiv. https://www.biorxiv.org/content/10.1101/2022.01.15.476444v2. 10.1016/j.isci.2022.104112 PMC8983340 35402870

[55] Neerukonda SN, Vassell R, Herrup R, Liu S, Wang T, Takeda K, Yang Y, Lin TL, Wang W, Weiss CD. 2021. Establishment of a well-characterized SARS-CoV-2 lentiviral pseudovirus neutralization assay using 293T cells with stable expression of ACE2 and TMPRSS2. PLoS One 16:e0248348. doi:10.1371/journal.pone.0248348. 33690649 PMC7946320

[56] Ianevski A, Giri AK, Aittokallio T. 2022. SynergyFinder 3.0: an interactive analysis and consensus interpretation of multidrug synergies across multiple samples. Nucleic Acids Res 50 (W1):W739–W743. doi:10.1093/nar/gkac382. 35580060 PMC9252834

[57] Ianevski A, Timonen S, Kononov A, Aittokallio T, Giri AK. 2020. SynToxProfiler: an interactive analysis of drug combination synergy, toxicity and efficacy. PLoS Comput Biol 16:e1007604. doi:10.1371/journal.pcbi.1007604. 32012154 PMC7018095

[58] Finch CL, Dyall J, Xu S, Nelson EA, Postnikova E, Liang JY, Zhou H, DeWald LE, Thomas CJ, Wang A, Xu X, Hughes E, Morris PJ, Mirsalis JC, Nguyen LH, Arolfo MP, Koci B, Holbrook MR, Hensley LE, Jahrling PB, Schmaljohn C, Johansen LM, Olinger GG, Schiffer JT, White JM. 2021. Formulation, stability, pharmacokinetic, and modeling studies for tests of synergistic combinations of orally available approved drugs against Ebola virus *in vivo*. Microorganisms 9:566. doi:10.3390/microorganisms9030566. 33801811 PMC7998926

[59] Cokol-Cakmak M, Bakan F, Cetiner S, Cokol M. 2018. Diagonal method to measure synergy among any number of drugs. J Vis Exp 2018:57713. doi:10.3791/57713. PMC6101960 29985330

[60] Gidari A, Sabbatini S, Schiaroli E, Bastianelli S, Pierucci S, Busti C, Comez L, Libera V, Macchiarulo A, Paciaroni A, Vicenti I, Zazzi M, Francisci D. 2022. The combination of molnupiravir with nirmatrelvir or GC376 has a synergic role in the inhibition of SARS-CoV-2 replication *in vitro*. Microorganisms 10:1475. doi:10.3390/microorganisms10071475. 35889194 PMC9323947

[61] Li P, Wang Y, Lavrijsen M, Lamers MM, de Vries AC, Rottier RJ, Bruno MJ, Peppelenbosch MP, Haagmans BL, Pan Q. 2022. SARS-CoV-2 Omicron variant is highly sensitive to molnupiravir, nirmatrelvir, and the combination. Cell Res 32:322–324. doi:10.1038/s41422-022-00618-w. 35058606 PMC8771185

[62] Jeong JH, Chokkakula S, Min SC, Kim BK, Choi W-S, Oh S, Yun YS, Kang DH, Lee O-J, Kim E-G, Choi J-H, Lee J-Y, Choi YK, Baek YH, Song M-S. 2022. Combination therapy with nirmatrelvir and molnupiravir improves the survival of SARS-CoV-2 infected mice. bioRxiv. https://www.biorxiv.org/content/10.1101/2022.06.27.497875v1. 10.1016/j.antiviral.2022.105430 PMC9535923 36209984

[63] Shapira T, Monreal IA, Dion SP, Buchholz DW, Imbiakha B, Olmstead AD, Jager M, Desilets A, Gao G, Martins M, Vandal T, Thompson CAH, Chin A, Rees WD, Steiner T, Nabi IR, Marsault E, Sahler J, Diel DG, Van de Walle GR, August A, Whittaker GR, Boudreault PL, Leduc R, Aguilar HC, Jean F. 2022. A TMPRSS2 inhibitor acts as a pan-SARS-CoV-2 prophylactic and therapeutic. Nature 605:340–348. doi:10.1038/s41586-022-04661-w. 35344983 PMC9095466

[64] Bai X, Buckle AM, Vladar EK, Janoff EN, Khare R, Ordway D, Beckham D, Fornis LB, Majluf-Cruz A, Fugit RV, Freed BM, Kim S, Sandhaus RA, Chan ED. 2022. Enoxaparin augments α-1-antitrypsin inhibition of TMPRSS2, a promising drug combination against COVID-19. Sci Rep 12:5207. doi:10.1038/s41598-022-09133-9. 35338216 PMC8953970

[65] Essalmani R, Jain J, Susan-Resiga D, Andreo U, Evagelidis A, Derbali RM, Huynh DN, Dallaire F, Laporte M, Delpal A, Sutto-Ortiz P, Coutard B, Mapa C, Wilcoxen K, Decroly E, Nq Pham T, Cohen EA, Seidah NG. 2022. Distinctive roles of furin and TMPRSS2 in SARS-CoV-2 infectivity. J Virol 96:e00128-22. doi:10.1128/jvi.00128-22. 35343766 PMC9044946

[66] Chupp G, Spichler-Moffarah A, Søgaard OS, Esserman D, Dziura J, Danzig L, Chaurasia R, Patra KP, Salovey A, Nunez A, May J, Astorino L, Patel A, Halene S, Wang J, Hui P, Patel P, Lu J, Li F, Gan G, Parziale S, Katsovich L, Desir GV, Vinetz JM. 2022. A phase 2 randomized, double-blind, placebo-controlled trial of oral camostat mesylate for early treatment of COVID-19 outpatients showed shorter illness course and attenuation of loss of smell and taste. medRxiv. https://www.medrxiv.org/search/Chupp%252BG%252C%252BSpichler-Moffarah%252BA%252C%252BS%25C3%25B8gaard%252BOS.

[67] Mahoney M, Damalanka VC, Tartell MA, Chung DH, Lourenco AL, Pwee D, Mayer Bridwell AE, Hoffmann M, Voss J, Karmakar P, Azouz NP, Klingler AM, Rothlauf PW, Thompson CE, Lee M, Klampfer L, Stallings CL, Rothenberg ME, Pohlmann S, Whelan SPJ, O’Donoghue AJ, Craik CS, Janetka JW. 2021. A novel class of TMPRSS2 inhibitors potently block SARS-CoV-2 and MERS-CoV viral entry and protect human epithelial lung cells. Proc Natl Acad Sci USA 118:e2108728118. doi:10.1073/pnas.2108728118. 34635581 PMC8694051

[68] Kreutzberger AJB, Sanyal A, Ojha R, Pyle JD, Vapalahti O, Balistreri G, Kirchhausen T. 2021. Synergistic block of SARS-CoV-2 infection by combined drug inhibition of the host entry factors PIKfyve kinase and TMPRSS2 protease. J Virol 95:e0097521. doi:10.1128/JVI.00975-21. 34406858 PMC8513479

[69] Chen SF, Ruben RL, Dexter DL. 1986. Mechanism of action of the novel anticancer agent 6-fluoro-2-(2′-fluoro-1,1′-biphenyl-4-yl)-3-methyl-4-quinolinecarboxylic acid sodium salt (NSC 368390): inhibition of de novo pyrimidine nucleotide biosynthesis. Cancer Res 46:5014–5019. 3019518

[70] Demarest JF, Kienle M, Boytz R, Ayres M, Kim EJ, Chung D, Gandhi V, Davey R, Sykes DB, Shohdy N, Pottage JC, Kumar VS. 2022. Brequinar and dipyridamole in combination exhibits synergistic antiviral activity against SARS-CoV-2 *in vitro*: rationale for a host-acting antiviral treatment strategy for COVID-19. bioRxiv. https://www.biorxiv.org/content/10.1101/2022.03.30.486499v2. 10.1016/j.antiviral.2022.105403 PMC9420051 36041646

[71] Goyal A, Duke ER, Cardozo-Ojeda EF, Schiffer JT. 2022. Modeling explains prolonged SARS-CoV-2 nasal shedding relative to lung shedding in remdesivir treated rhesus macaques. iScience 25:104448. doi:10.1016/j.isci.2022.104448. 35634576 PMC9130309

[72] Nguyenla X, Wehri E, Van Dis E, Biering SB, Yamashiro LH, Stroumza J, Dugast-Darzacq C, Graham T, Stanley S, Schaletzky J. 2020. Discovery of SARS-CoV-2 antiviral synergy between remdesivir and approved drugs in human lung cells. bioRxiv. https://www.biorxiv.org/content/10.1101/2020.09.18.302398v1. 10.1038/s41598-022-21034-5 PMC9628577 36323770

[73] Mahase E. 2021. Covid-19: molnupiravir reduces risk of hospital admission or death by 50% in patients at risk, MSD reports. BMJ 375:n2422. doi:10.1136/bmj.n2422. 34607801

[74] Mahase E. 2021. Covid-19: Pfizer’s paxlovid is 89% effective in patients at risk of serious illness, company reports. BMJ 375:n2713. doi:10.1136/bmj.n2713. 34750163

[75] Moghadasi SA, Heilmann E, Moraes SN, Kearns FL, von Laer D, Amaro RE, Harris RS. 2022. Transmissible SARS-CoV-2 variants with resistance to clinical protease inhibitors. bioRxiv. https://www.biorxiv.org/content/10.1101/2022.08.07.503099v1. 10.1126/sciadv.ade8778 PMC10058310 36989354

[76] Hu Y, Lewandowski EM, Tan H, Morgan RT, Zhang X, Jacobs LMC, Butler SG, Mongora MV, Choy J, Chen Y, Wang J. 2022. Naturally occurring mutations of SARS-CoV-2 main protease confer drug resistance to nirmatrelvir. bioRxiv. https://www.biorxiv.org/content/10.1101/2022.06.28.497978v2. 10.1021/acscentsci.3c00538 PMC10451032 37637734

[77] Szemiel AM, Merits A, Orton RJ, MacLean OA, Pinto RM, Wickenhagen A, Lieber G, Turnbull ML, Wang S, Furnon W, Suarez NM, Mair D, da Silva Filipe A, Willett BJ, Wilson SJ, Patel AH, Thomson EC, Palmarini M, Kohl A, Stewart ME. 2021. *In vitro* selection of remdesivir resistance suggests evolutionary predictability of SARS-CoV-2. PLoS Pathog 17:e1009929. doi:10.1371/journal.ppat.1009929. 34534263 PMC8496873

[78] Checkmahomed L, Carbonneau J, Du Pont V, Riola NC, Perry JK, Li J, Pare B, Simpson SM, Smith MA, Porter DP, Boivin G. 2022. *In vitro* selection of remdesivir-resistant SARS-CoV-2 demonstrates high barrier to resistance. Antimicrob Agents Chemother 66:e00198-22. doi:10.1128/aac.00198-22. 35708323 PMC9295571

[79] Hogan JI, Duerr Dimartino D, Marier C, Hochman S, Mehta S, Wang G, Heguy A. 2022. Remdesivir resistance in transplant recipients with persistent COVID-19. Res Sq doi:10.21203/rs.3.rs-1800050/v1. PMC9619446 36156117

[80] Martinez DR, Schafer A, Leist SR, Li D, Gully K, Yount B, Feng JY, Bunyan E, Porter DP, Cihlar T, Montgomery SA, Haynes BF, Baric RS, Nussenzweig MC, Sheahan TP. 2021. Prevention and therapy of SARS-CoV-2 and the B.1.351 variant in mice. Cell Rep 36:109450. doi:10.1016/j.celrep.2021.109450. 34289384 PMC8270748

[81] Jilek BL, Zarr M, Sampah ME, Rabi SA, Bullen CK, Lai J, Shen L, Siliciano RF. 2012. A quantitative basis for antiretroviral therapy for HIV-1 infection. Nat Med 18:446–451. doi:10.1038/nm.2649. 22344296 PMC3296892

