# Supplementary material for: Combinations of Host- and Virus-Targeting Antiviral Drugs Confer Synergistic Suppression of SARS-CoV-2
Source: Microbiol Spectr. 2022 Oct 3;10(5):e03331-22. doi: 10.1128/spectrum.03331-22 (PMC9718484; doi:10.1128/spectrum.03331-22)
Supplement: Supplemental file 1 — Table S1, Table S2, Fig. S1-S6. Download spectrum.03331-22-s0001.pdf, PDF file, 1.6 MB [file spectrum.03331-22-s0001.pdf]

SUPPLEMENTAL FIGURES & TABLES

Table S1: Compounds evaluated in this study.

Compound	Vendor	Catalog number
Entry Inhibitors		
Apilimod	Axon Medchem	1369
Arbidol	Commercial	reference(1)
Avoralstat	MedChemExpress	HY-16735
Camostat Mesylate	Sigma Aldrich	SML0057
Imatinib Mesylate	TCI America	I0936
Nafamostat Mesylate	TCI America	N0959
Replication Inhibitors		
Brequinar	MedChemExpress	HY-108325
EIDD-1931 (cell active form of molnupiravir)	MedChemExpress	HY-125033
Nirmatrelvir (PF-07321332)	MedChemExpress	HY-138687

Compound	Oral C_{max} (µM)	IC_{50} (µM) (Literature)	Oral C_{max}/IC_{50} (Literature)	IC_{50} (µM) (This Study)	Oral C_{max}/IC_{50} (This Study)
Entry Inhibitors					
Apilimod	0.265	**4.5**	0.06	41.69	0.11
Arbidol	~1.0	nd	nd	20	0.05
Avoralstat	0.58	2.8	0.21	**2.6**	**0.22**
Camostat	0.278	0.11	2.53	**0.726**	**0.38**
Imatinib	6.85	4.86	1.41	60	0.11
Nafamostat	na	0.005	na	**0.007**	na
Replication Inhibitors					
Brequinar	66.6	**~2**	~**33.3**	**50**	**1.33**
Molnupiravir	14	**0.08**	**175**	**0.15**	**93.3**

Table S2. Solo agent efficacy and human exposure of drugs tested in combinations vs. SARS-CoV-2 infection of Calu-3 cells. IC_{50} values in **bold** are from studies of authentic SARS-CoV-2 infections in Calu-3 cells or (for imatinib) in lung organoids. IC_{50} values in column 3 are from the literature: apilimod (2); avoralstat (3); camostat (4); imatinib (5); nafamostat (6); brequinar (7); molnupiravir (8). IC_{50} values in column 5 are from this study using pseudoviruses for apilimod, arbidol and imatinib, and authentic SARS-2 WA-1 for brequinar, camostat and molnupiravir. All tests were done in Calu-3 cells. For pseudovirus studies, vesicular stomatitis virus (VSV) pseudotyped with the Spike glycoprotein (GP) from SARS-CoV-2 were prepared and used as follows. BHK21 cells were transfected with a plasmid containing the full-length, codon optimized SARS-CoV-2 GP sequence pCG1-SARS-2-S (full length), which is based on the NCBI Reference Sequence: YP_009724390.1, using X-tremeGENE 9 DNA Transfection Reagent (Roche Applied Science; cat. 06 365 787 001). The following day, cells were infected with VSV-G helper virus –VSVΔG-Renilla (Kerafast, EH1020-PM). Twenty-four hours later, the VSV-Spike pseudovirus (PV) preparation was cleared of cellular debris by centrifugation and stored at -80°C. For experiments with apilimod, arbidol and imatinib, compounds were added to plates containing Calu-3 cells, followed immediately by addition of VSV-Spike pseudovirus to each infected well or virus-free medium to each uninfected well. Plates were incubated at 37°C, 5% CO_2 for 24 hours. The readout for pseudovirus experiments was Renilla luciferase (Promega), performed as described (9). Human oral C_{max} data in column 2 are from: apilimod (10), arbidol (11-13), avoralstat (14); camostat (Package Insert); imatinib (15); brequinar (16); molnupiravir (17). In all studies, molnupiravir was provided as the active form, EIDD-1931. Apilimod is an oral pre-clinical (Ph2) PIKfyve inhibitor. Arbidol is an approved oral fusion inhibitor. Avoralstat is an oral pre-clinical (Ph3) TMPRSS2 inhibitor. Camostat is an approved oral TMPRSS2 inhibitor. Imatinib is an approved oral Abl kinase inhibitor. Nafamostat is an approved inhaled TMPRSS2 inhibitor. Brequinar is an oral pre-clinical (Ph 2) inhibitor of dihydroorotate dehydrogenase (DHODH). Molnupiravir is an approved oral SARS-CoV-2 Polymerase inhibitor. nd, not done. na, not available.

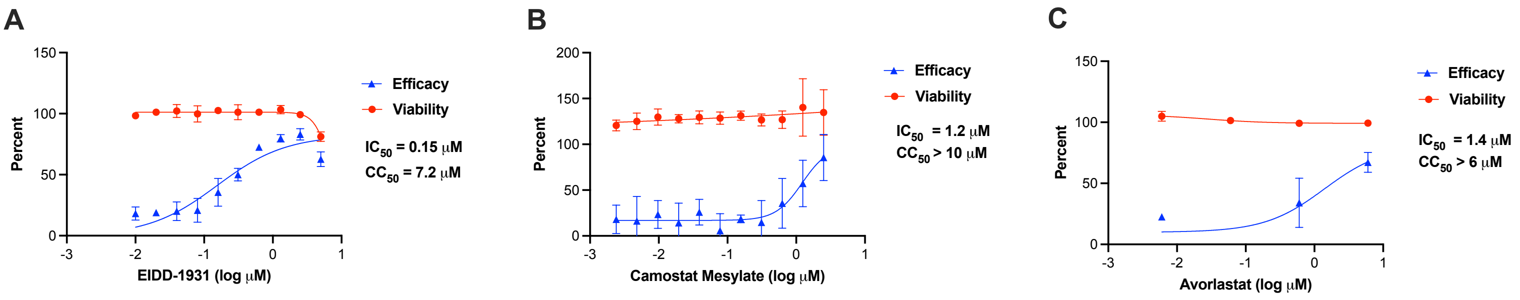

Figure S1: Antiviral efficacy of a DAA and HTAs in 293TAT cells. Cells were treated with the indicated concentrations of drugs for 2 hours prior to infection with SARS-CoV-2 WA1 at a MOI of 0.05. Parallel plates contained cells treated only with drugs. Forty-eight hours post infection, cell viability was assessed using CellTiter-Glo assay and antiviral efficacy and viability were calculated as described in the Materials and Methods. Data points reflect average and standard deviations of triplicate samples per condition and curves were generated by nonlinear regression using Prism.

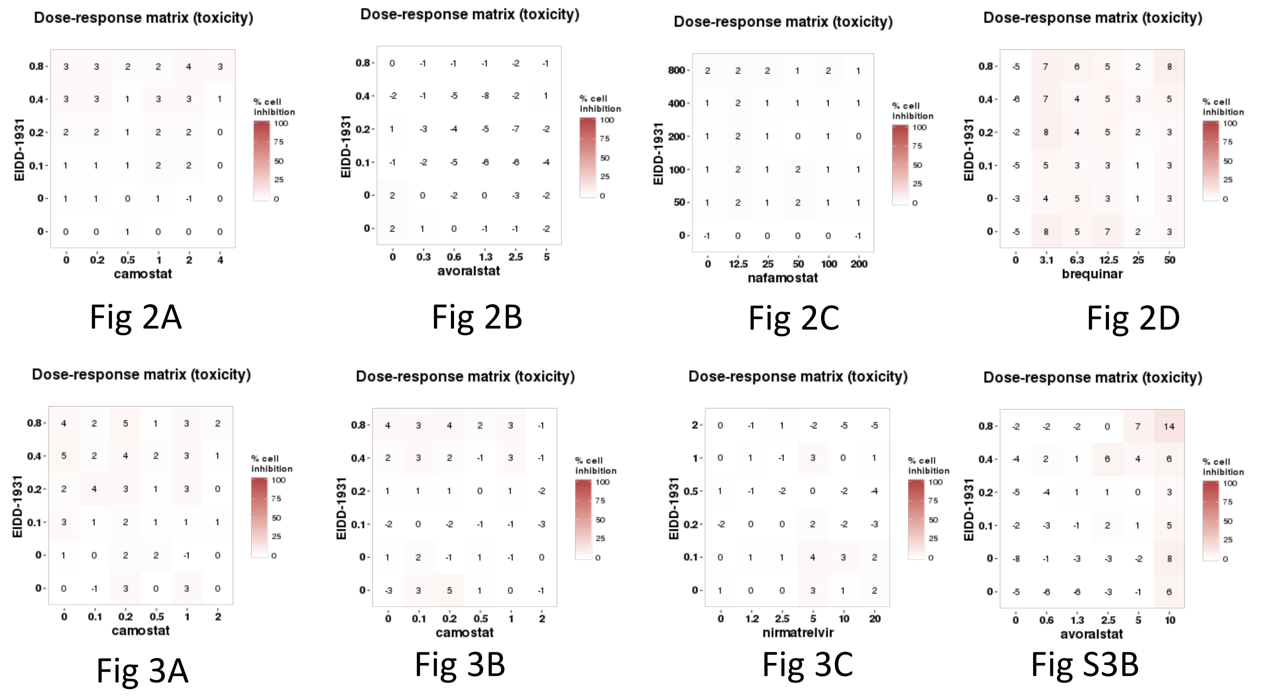

Figure S2: Drug combinations are not toxic to Calu-3 or 293TAT cells. Calu-3 or 293TAT cells were treated with the indicated drug concentrations and incubated for 48 (293TAT) or 96 (Calu-3) hours before cell viability was measured by CellTiter-Glo assay. Toxicity values were analyzed by SynToxProfiler.

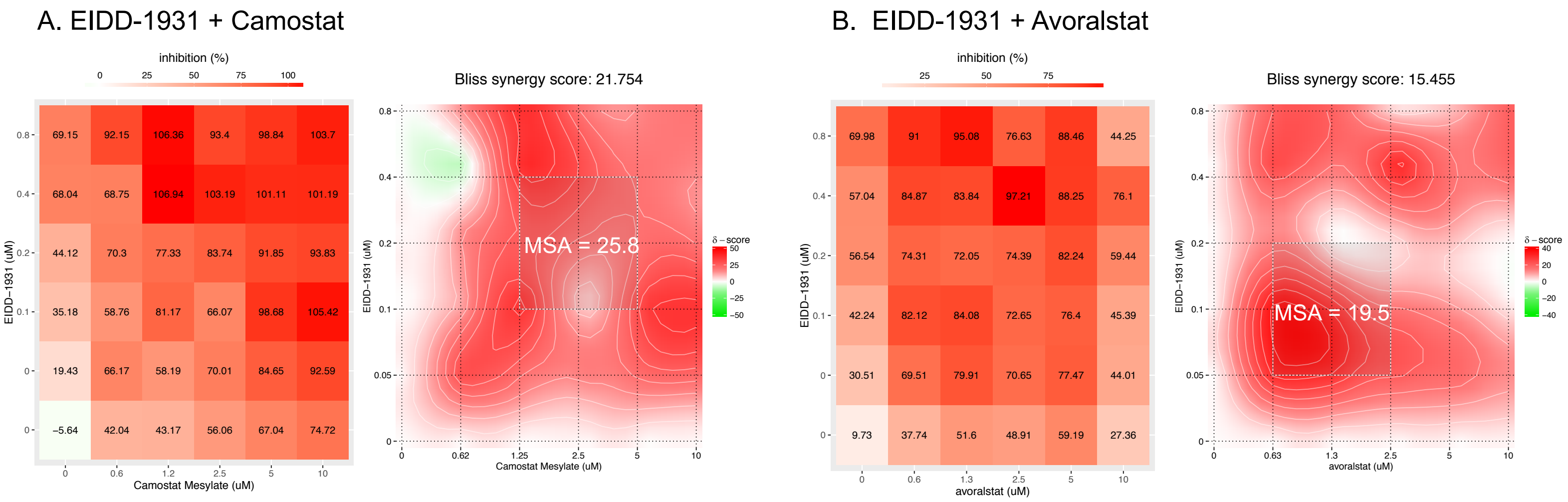

Figure S3: TMPRSS2 inhibitors synergize with molnupiravir to suppress SARS-CoV-2 in 293TAT cells. 293TAT cells were treated with the indicated concentrations of camostat, avoralstat, and molnupiravir (EIDD-1931) for two hours prior to infection with SARS-CoV-2 WA1 (moi 0.05). Forty-eight hours later, cell viability was measured by CellTiter-Glo assay and antiviral efficacy calculated as described in the Materials and Methods. For each panel A and B, the left plots show percent inhibition of infection, while the right plots depict 2-D topographs that highlight the areas of synergy across the dose response matrix, including the maximum synergistic area (MSA), which is designated with a light gray box.

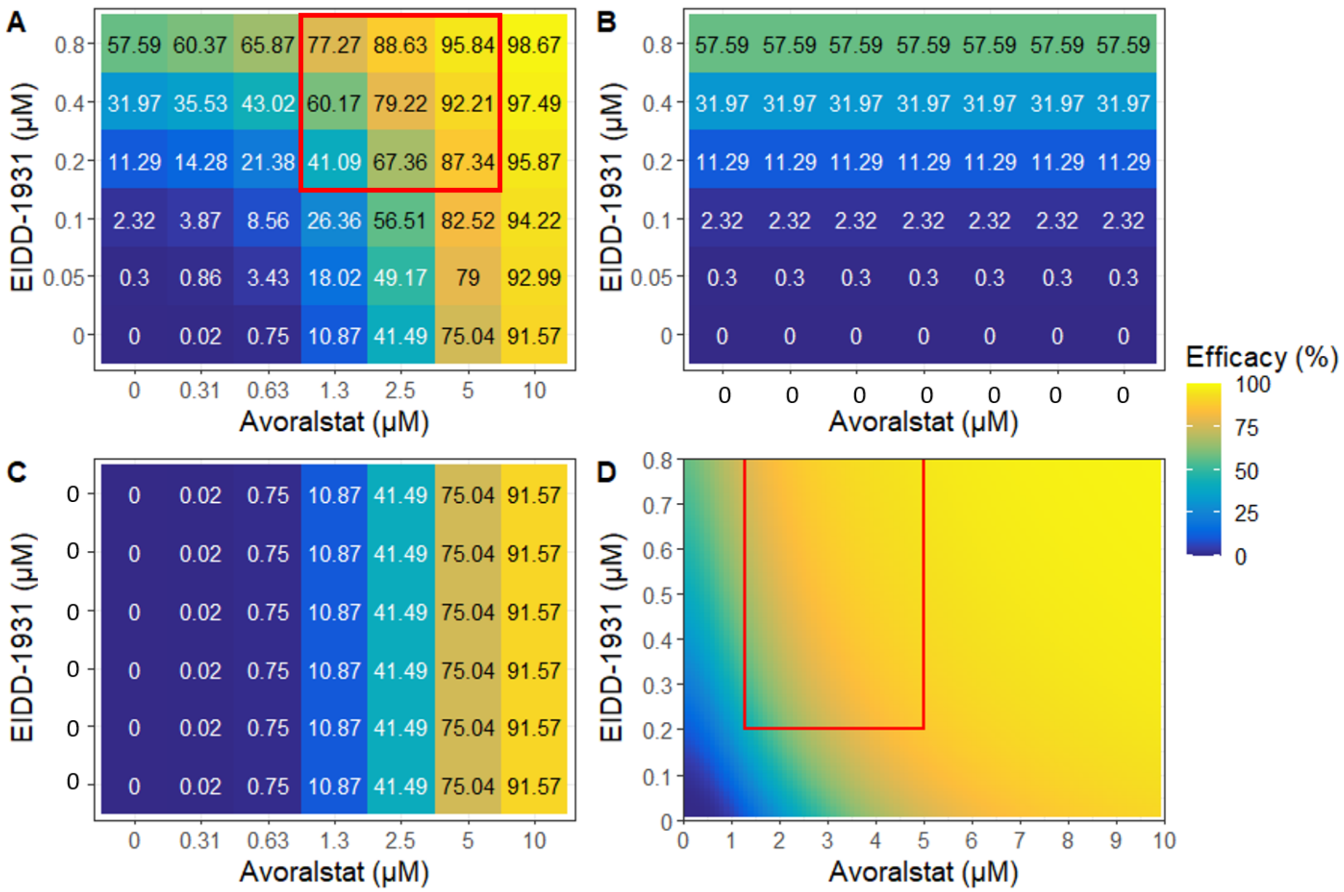

Figure S4: PD modeling of molnupiravir + avoralstat. A) Model projected efficacy of EIDD-1931 plus avoralstat at empirically tested concentrations. B) Projected efficacy of EIDD-1931 alone. C) Projected efficacy of avoralstat alone. D) Heat map of model projected inhibition at all combinatorial concentrations of both agents. Red box denotes the maximum synergistic area (MSA).

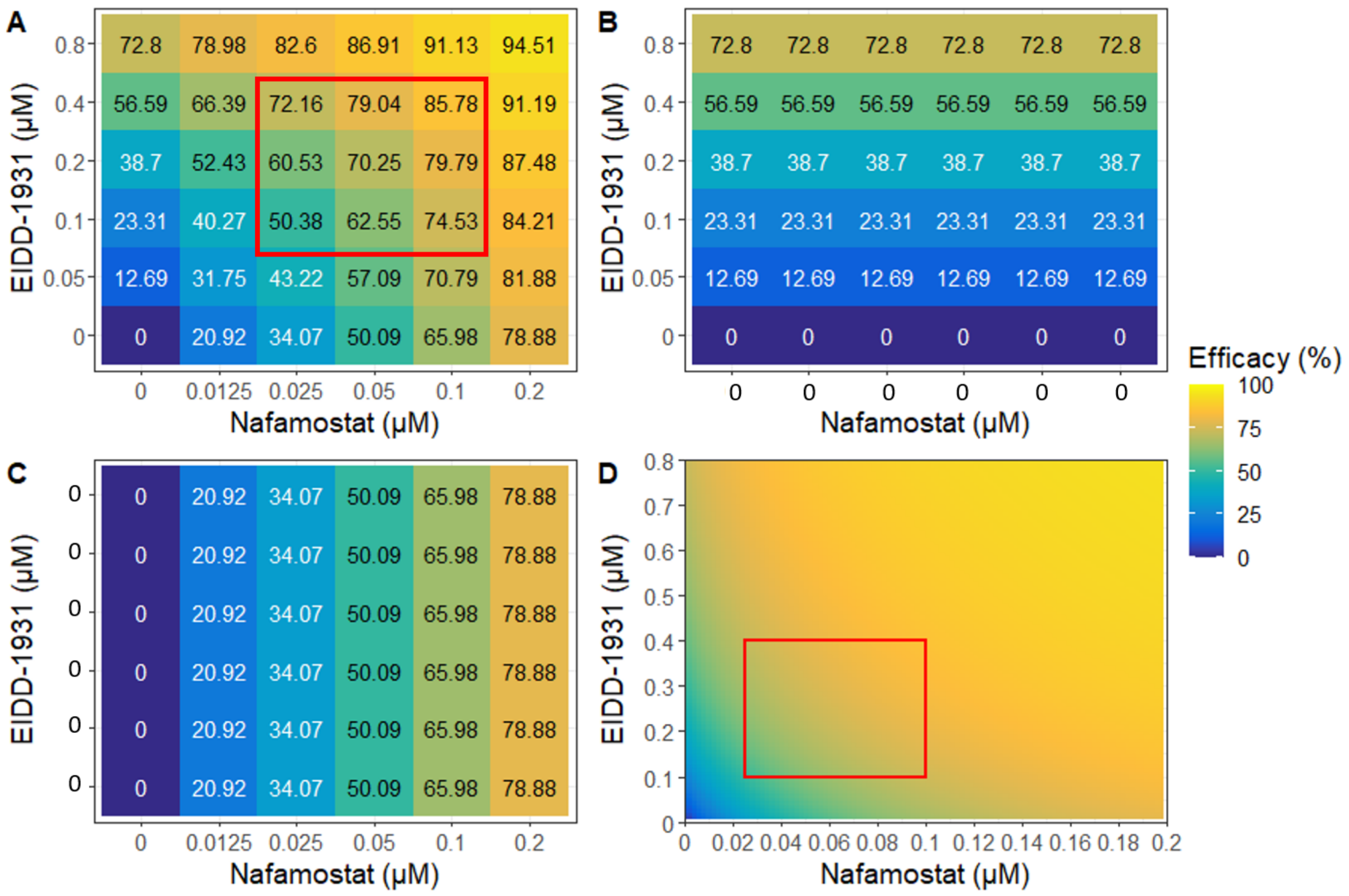

Figure S5: PD modeling of molnupiravir + nafamostat. A) Model projected efficacy of EIDD-1931 plus nafamostat at empirically tested concentrations. B) Projected efficacy of EIDD-1931 alone. C) Projected efficacy of nafamostat alone. D) Heat map of model projected inhibition at all combinatorial concentrations of both agents. Red box denotes the maximum synergistic area (MSA).

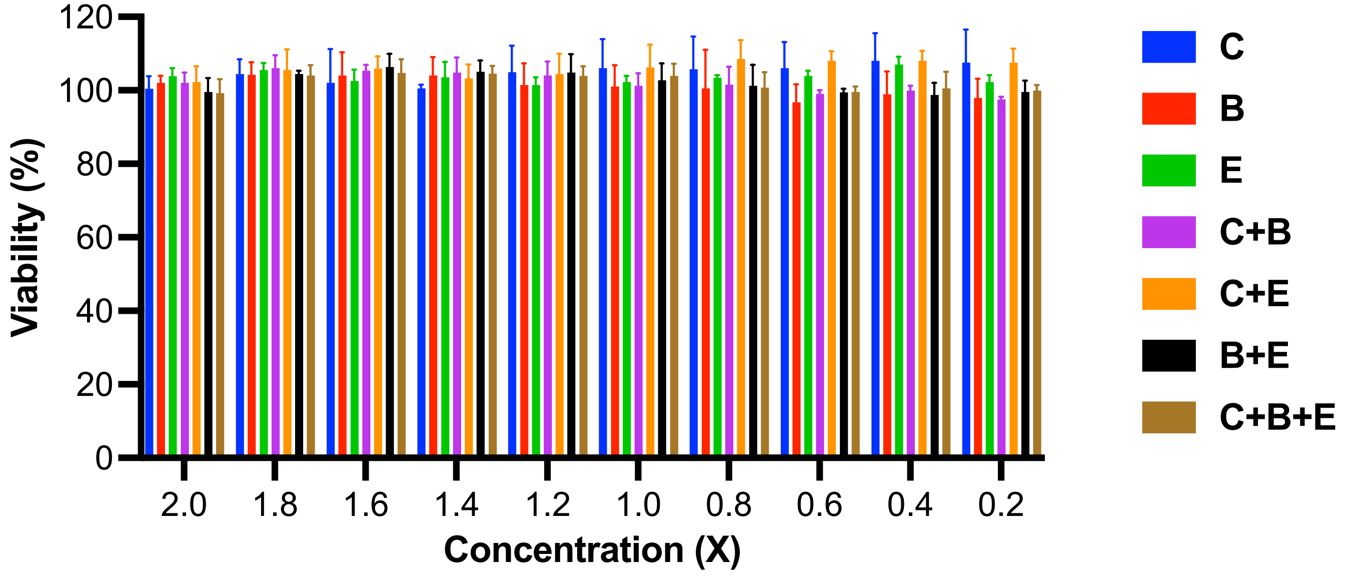

Figure S6: Highly potent double and triple antiviral combinations of host-targeting (HTA) and viral-targeting (DAA) drugs are not toxic to cells. Camostat (C), brequinar (B), and molnupiravir (E) were mixed at top 2.0X concentrations of 1.4μM, 50μM, and 0.28μM, respectively to produce 1-, 2- or 3-drug combinations. "Concentration (X)" on the x-axis refers to the concentration of drug(s) in mixture based on the top 2.0X concentrations. Stock concentrations were serially diluted in 10% increments and added to Calu-3 cells. Ninety-six hours later, cell viability was measured by CellTiter-Glo assay. Data reflect average and standard deviation of triplicate samples per condition, from a single experiment, which was conducted twice with similar results.

REFERENCES

1. Pecheur EI, Borisevich V, Halfmann P, Morrey JD, Smee DF, Prichard M, Mire CE, Kawaoka Y, Geisbert TW, Polyak SJ. 2016. The Synthetic Antiviral Drug Arbidol Inhibits Globally Prevalent Pathogenic Viruses. J Virol 90:3086-92.
2. Dittmar M, Lee JS, Whig K, Segrist E, Li M, Kamalia B, Castellana L, Ayyanathan K, Cardenas-Diaz FL, Morrisey EE, Truitt R, Yang W, Jurado K, Samby K, Ramage H, Schultz DC, Cherry S. 2021. Drug repurposing screens reveal cell-type-specific entry pathways and FDA-approved drugs active against SARS-Cov-2. Cell Rep 35:108959.
3. Sun YJ, Velez G, Parsons DE, Li K, Ortiz ME, Sharma S, McCray PB, Jr., Bassuk AG, Mahajan VB. 2021. Structure-based phylogeny identifies avoralstat as a TMPRSS2 inhibitor that prevents SARS-CoV-2 infection in mice. J Clin Invest 131.
4. Hoffmann M, Hofmann-Winkler H, Smith JC, Kruger N, Arora P, Sorensen LK, Sogaard OS, Hasselstrom JB, Winkler M, Hempel T, Raich L, Olsson S, Danov O, Jonigk D, Yamazoe T, Yamatsuta K, Mizuno H, Ludwig S, Noe F, Kjolby M, Braun A, Sheltzer JM, Pohlmann S. 2021. Camostat mesylate inhibits SARS-CoV-2 activation by TMPRSS2-related proteases and its metabolite GBPA exerts antiviral activity. EBioMedicine 65:103255.

5. Han Y, Duan X, Yang L, Nilsson-Payant BE, Wang P, Duan F, Tang X, Yaron TM, Zhang T, Uhl S, Bram Y, Richardson C, Zhu J, Zhao Z, Redmond D, Houghton S, Nguyen DT, Xu D, Wang X, Jessurun J, Borczuk A, Huang Y, Johnson JL, Liu Y, Xiang J, Wang H, Cantley LC, tenOever BR, Ho DD, Pan FC, Evans T, Chen HJ, Schwartz RE, Chen S. 2021. Identification of SARS-CoV-2 inhibitors using lung and colonic organoids. Nature 589:270-275.
6. Hoffmann M, Schroeder S, Kleine-Weber H, Muller MA, Drosten C, Pohlmann S. 2020. Nafamostat Mesylate Blocks Activation of SARS-CoV-2: New Treatment Option for COVID-19. Antimicrob Agents Chemother 64.
7. Schultz DC, Johnson RM, Ayyanathan K, Miller J, Whig K, Kamalia B, Dittmar M, Weston S, Hammond HL, Dillen C, Ardanuy J, Taylor L, Lee JS, Li M, Lee E, Shoffler C, Petucci C, Constant S, Ferrer M, Thaiss CA, Frieman MB, Cherry S. 2022. Pyrimidine inhibitors synergize with nucleoside analogues to block SARS-CoV-2. Nature doi:10.1038/s41586-022-04482-x.
8. Sheahan TP, Sims AC, Zhou S, Graham RL, Pruijssers AJ, Agostini ML, Leist SR, Schafer A, Dinnon KH, 3rd, Stevens LJ, Chappell JD, Lu X, Hughes TM, George AS, Hill CS, Montgomery SA, Brown AJ, Bluemling GR, Natchus MG, Saindane M, Kolykhalov AA, Painter G, Harcourt J, Tamin A, Thornburg NJ, Swanstrom R, Denison MR, Baric RS. 2020. An orally bioavailable broad-spectrum antiviral inhibits SARS-CoV-2 in human airway epithelial cell cultures and multiple coronaviruses in mice. Sci Transl Med 12.
9. Herring S, Oda JM, Wagoner J, Kirchmeier D, O'Connor A, Nelson EA, Huang Q, Liang Y, DeWald LE, Johansen LM, Glass PJ, Olinger GG, Ianevski A, Aittokallio T, Paine MF, Fink SL, White JM, Polyak SJ. 2021. Inhibition of Arenaviruses by Combinations of Orally Available Approved Drugs. Antimicrob Agents Chemother 65.
10. Wada Y, Cardinale I, Khatcherian A, Chu J, Kantor AB, Gottlieb AB, Tatsuta N, Jacobson E, Barsoum J, Krueger JG. 2012. Apilimod inhibits the production of IL-12 and IL-23 and reduces dendritic cell infiltration in psoriasis. PLoS One 7:e35069.
11. Sun Y, He X, Qiu F, Zhu X, Zhao M, Li-Ling J, Su X, Zhao L. 2013. Pharmacokinetics of single and multiple oral doses of arbidol in healthy Chinese volunteers. Int J Clin Pharmacol Ther 51:423-32.
12. Deng P, Zhong D, Yu K, Zhang Y, Wang T, Chen X. 2013. Pharmacokinetics, metabolism, and excretion of the antiviral drug arbidol in humans. Antimicrob Agents Chemother 57:1743-55.
13. Liu MY, Wang S, Yao WF, Wu HZ, Meng SN, Wei MJ. 2009. Pharmacokinetic properties and bioequivalence of two formulations of arbidol: an open-label, single-dose, randomized-sequence, two-period crossover study in healthy Chinese male volunteers. Clin Ther 31:784-92.
14. Cornpropst M, Collis P, Collier J, Babu YS, Wilson R, Zhang J, Fang L, Zong J, Sheridan WP. 2016. Safety, pharmacokinetics, and pharmacodynamics of avoralstat, an oral plasma kallikrein inhibitor: phase 1 study. Allergy 71:1676-1683.
15. Peng B, Lloyd P, Schran H. 2005. Clinical pharmacokinetics of imatinib. Clin Pharmacokinet 44:879-94.
16. Joshi AS, King SY, Zajac BA, Makowka L, Sher LS, Kahan BD, Menkis AH, Stiller CR, Schaefle B, Kornhauser DM. 1997. Phase I safety and pharmacokinetic studies of brequinar sodium after single ascending oral doses in stable renal, hepatic, and cardiac allograft recipients. J Clin Pharmacol 37:1121-8.

17. Painter WP, Holman W, Bush JA, Almazedi F, Malik H, Eraut N, Morin MJ, Szewczyk LJ, Painter GR. 2021. Human Safety, Tolerability, and Pharmacokinetics of Molnupiravir, a Novel Broad-Spectrum Oral Antiviral Agent with Activity Against SARS-CoV-2. Antimicrob Agents Chemother doi:10.1128/AAC.02428-20.
